# Outbreaks of Fungal Infections in Hospitals: Epidemiology, Detection, and Management

**DOI:** 10.3390/jof9111059

**Published:** 2023-10-29

**Authors:** Abby P. Douglas, Adam G. Stewart, Catriona L. Halliday, Sharon C.-A. Chen

**Affiliations:** 1 National Centre for Infections in Cancer, Peter MacCallum Cancer Centre, Melbourne, VIC 3000, Australia; 2 Department of Infectious Diseases, Peter MacCallum Cancer Centre, Melbourne, VIC 3000, Australia; 3 Sir Peter MacCallum Department of Oncology, University of Melbourne, Melbourne, VIC 3000, Australia; 4 Department of Infectious Diseases, Austin Health, Heidelberg, VIC 3084, Australia; 5 Centre for Clinical Research, Faculty of Medicine, Royal Brisbane and Women’s Hospital Campus, The University of Queensland, Herston, QLD 4006, Australia; adam.stewart@health.qld.gov.au; 6 Centre for Infectious Diseases and Microbiology Laboratory Services, Institute of Clinical Pathology and Medical Research, New South Wales Health Pathology, Westmead Hospital, Sydney, NSW 2145, Australia; catriona.halliday@health.nsw.gov.au (C.L.H.); sharon.chen@health.nsw.gov.au (S.C.-A.C.); 7 Sydney Medical School, Faculty of Medicine and Health, The University of Sydney, Sydney, NSW 2050, Australia

**Keywords:** fungi, nosocomial, outbreak, whole genome sequencing, genotyping, yeast, mould, infection prevention

## Abstract

Nosocomial clusters of fungal infections, whilst uncommon, cannot be predicted and are associated with significant morbidity and mortality. Here, we review reports of nosocomial outbreaks of invasive fungal disease to glean insight into their epidemiology, risks for infection, methods employed in outbreak detection including genomic testing to confirm the outbreak, and approaches to clinical and infection control management. Both yeasts and filamentous fungi cause outbreaks, with each having general and specific risks. The early detection and confirmation of the outbreak are essential for diagnosis, treatment of affected patients, and termination of the outbreak. Environmental sampling, including the air in mould outbreaks, for the pathogen may be indicated. The genetic analysis of epidemiologically linked isolates is strongly recommended through a sufficiently discriminatory approach such as whole genome sequencing or a method that is acceptably discriminatory for that pathogen. An analysis of both linked isolates and epidemiologically unrelated strains is required to enable genetic similarity comparisons. The management of the outbreak encompasses input from a multi-disciplinary team with epidemiological investigation and infection control measures, including screening for additional cases, patient cohorting, and strict hygiene and cleaning procedures. Automated methods for fungal infection surveillance would greatly aid earlier outbreak detection and should be a focus of research.

## 1. Introduction

Invasive fungal diseases (IFDs) are recognized as a global health concern by the World Health Organization (https://www.who.int/publications/i/item/9789240060241 [accessed on 7 July 2023]) and are caused by an increasing breadth of yeast, filamentous fungi, and dimorphic fungi pathogens. As fungi are ubiquitous in nature, clusters of infection can and have occurred, not only in the community but as nosocomial infection when there is transmission or acquisition of fungi in the healthcare environment. Some yeast pathogens, such as *Candida* spp., tend to colonize the skin and gastrointestinal and genitourinary systems in normal hosts and have not often been as readily recognized as nosocomial pathogens as moulds. However, with the advent of *Candida auris* infections and with more sophisticated pathogen identification and typing methods, the ability of other *Candida* and yeast species such as *Candida parapsilosis* and *Candida tropicalis* to colonize the hospital environment and effect patient-to-patient spread with potentially serious consequences has been highlighted. Significant notable outbreaks due to mould species have also occurred in relation to contaminated medical products, and hospital construction or renovations poses a high-risk period. The aim of this work is to review reports of nosocomial outbreaks of pathogenic fungi to gain insights into methods employed in the diagnosis of infection and in outbreak detection, genomic testing to confirm an outbreak, and approaches to clinical and infection control management of case clusters. For the purposes of the present review, a potential outbreak is defined as ≥2 cases of a particular fungal infection linked in time and/or place, where the fungal infection was not evident on admission and was diagnosed and likely acquired in hospital. 

## 2. Diagnosis, Detection, and Genomic Tracking of Outbreaks

Before describing the epidemiology of individual fungal outbreaks and results thereof, the methods for diagnosis of fungal infections are broadly outlined as are the general principles of genomic testing for outbreak investigation. 

The detection of fungal outbreaks requires a multi-disciplinary approach between clinical microbiologists, epidemiologists, and public health units. The diagnostic mycology laboratory is key in (i) verifying the diagnosis and (ii) confirming the source of the outbreak [1]. Culture-based methods of specimens remain the cornerstone of diagnosis, although culture-independent methods are increasingly utilized [2]. Whilst the latter are important for diagnosis, culture is still essential for fungal isolation, drug susceptibility testing, and epidemiological comparisons [3]. For detailed descriptions of each of the methods, the reader is referred to recent reviews on the topic [1,2,3,4,5,6].

### 2.1. Direct Examination and Histopathology

Fungal stains such as periodic acid Schiff (PAS) and Grocott methenamine silver (GMS) used in histopathological examination or direct examination of clinical specimens provide clues to the presence of a fungal infection. Within tissue, hyphae may be: (i) hyaline septate, e.g., *Aspergillus*, *Fusarium* species; (ii) broad ribbon-like with rare septation which includes members of the order Mucorales; and (iii) pigmented or dematiceous, e.g., *Cladophialophora*, *Exophiala*, spp. [7]. However, the diagnostic accuracy of identification to genus or species level is less than 80% [8,9]. The most common cause for incorrect histopathologic diagnoses is the misidentification of Mucorales and *Aspergillus* spp. [10]. However, misidentifications can also occur with yeast infection, e.g., between *Histoplasma capsulatum* and *Nakaseomyces* (previously *Candida) glabratus*. It is essential that histopathological results are correlated with microbiology [9,10,11]. 

*Pneumocystis jirovecii* is non-culturable, and its diagnosis has traditionally relied upon the visualization of cyst or trophozoite forms in tissue, induced sputum (IS), or bronchoalveolar lavage (BAL) fluid specimens using optical brighteners, silver stains, and toluidine blue [12]. Immunofluorescence assays offer superior sensitivity (>90%) to conventional microscopy (50–80%) [13], with the diagnostic yield highest in HIV-infected patients [14,15].

### 2.2. Culture

Although slow, fungal culture remains the gold standard for diagnosing IFD. In the case of invasive *Candida* infections, blood cultures are estimated to identify only approximately 50% of all patients due to the rapid clearance of viable organisms from the bloodstream [16]. Although moulds such as *Fusarium*, *Aspergillus* grow easily on routine mycological media, recovery from clinical specimens of the Mucorales is difficult and is positive in only 15–25% of cases [17]. Chromogenic agar media, such as CHROMagar™ Candida (CHROMagar, Paris, France), are widely used in clinical laboratories for the isolation and presumptive identification of common *Candida* species, and more recently, CHROMagar™ Candida Plus was developed to specifically identify *C. auris* [18]. This medium improves the recovery of *C. auris* from surveillance swabs compared with conventional isolation media whilst also obviating the need for confirmatory identification tests [18,19]. Additionally, a 10% salt Sabouraud dulcitol enrichment broth was developed for the isolation of *C. auris* in patient and environmental specimens [20]. 

### 2.3. Nucleic Acid Detection

Either broad-range (or panfungal) assays for capturing “all fungi” or assays tailored to detect a specific genus/species may be used to directly detect fungal DNA in clinical specimens [2]. Panfungal PCR followed by DNA sequencing allows for the identification of the pathogen from a diverse range of clinical specimens, including formalin fixed paraffin embedded (FFPE) tissue, although the examination of the latter is only recommended where fungal forms are seen on histopathology [2,7,11]. One study reported results for blood, cerebrospinal fluid (CSF), and aspirates where the sensitivity, specificity, negative predictive value (NPV), and positive predictive value (PPV) were 100, 96, 100, and 86%, respectively. However, these values decreased to 90, 75, 86, and 82%, respectively, for BAL fluid specimens [21]. Such assays are particularly helpful for the detection of unexpected fungal pathogens for which fungus-targeted assays may not exist at the time of testing, e.g., the investigation of the outbreak of *Exserohilum rostratum* meningitis [22,23] (see Section 3.2.5).

For either screening for, or confirmation of, invasive aspergillosis (IA), the detection of *Aspergillus* using PCR has a sensitivity and specificity of 79% and 80% using blood, respectively [24]. On BAL fluid, performance improves when PCR is combined with antigen-based biomarkers [25] (see below). The direct detection of Mucorales DNA in fresh and FFPE tissue and BAL fluid specimens is helpful for species identification [26,27], and real-time quantitative PCR (qPCR) has also been used to detect Mucorales DNA in serum [26,28]. During the outbreak of *E. rostratum* meningitis, species-specific real-time PCR assays provided laboratory confirmation of 47% of case patients compared with culture and conventional PCR (14% and 29%, respectively) [22,29].

For pneumocystis pneumonia (PCP), qPCR is increasingly used instead of microscopy to detect *P. jirovecii* DNA in respiratory specimens [14], with qPCR now included by the European Organization for Research and Treatment of Cancer-Mycoses Study Group Education and Research Consortium (EORTC-MSGERC) as evidence of probable disease [12]. PCR-based assays are also often used for screening exposed patients during outbreaks and to assist source determination [30,31,32]. Whilst the quantitation of copy numbers of *P. jirovecii* DNA or assessment of burden through the cycle threshold (Ct) cutoff value may be useful in distinguishing infection from colonization with *P. jirovecii*, these “cutoffs” are variable and are dependent on the PCR platform, gene tested, and the patient population, e.g., HIV-positive vs. HIV-negative patients, due to known differences in fungal burden [14].

The position of *Candida* PCR assays in the routine diagnosis of invasive candidiasis (IC) remains uncertain. Commercial assays lack standardized methodologies despite showing a pooled sensitivity and specificity of 95% and 92%, respectively (compared with culture, where sensitivity and specificity are 85% and 38%, respectively) [33]. Only the T2Candida magnetic resonance assay panel (T2 Biosystems, Lexington, MA, USA) has been cleared by the US Food and Drug Administration (FDA) for the detection of five common Candida species in whole blood (sensitivity of 91.1%; time to positivity, 4.4 +/− 1 h) [34,35]. Prompt identification of individuals infected or colonized by *C. auris* is essential for minimizing cross infections in hospital outbreaks (see below). In-house and commercial PCR assays are available for the detection of *C. auris* from surveillance samples. These assays yield results within 1–4 h compared to 4–14 days for a culture [36]. Commercial assays include the Auris ID (OLM Diagnostics, Newcastle Upon Tyne, UK), Fungiplex (Bruker, Bremen, Germany) and the T2Cauris panel (T2 Biosystems, Lexington, MA, USA) [36].

### 2.4. Immunodiagnostic Assays

The main biomarker for the diagnosis of IA is *Aspergillus* galactomannan (GM) (Platelia *Aspergillus* Ag, Bio-Rad, Marnes-La-Coquette, France). Results obtained using serum and plasma have shown moderate-to-good pooled sensitivities and specificities (0.48–0.92 and 0.85–0.95, respectively) [37,38], with a similar performance on BAL fluid (0.61–0.92 and 0.81–0.98, respectively) [38,39,40]. For cementing a diagnosis, testing on BAL fluid is preferred. Cross reactive positive GM has been reported in patients with *Fusarium*, *Penicillium* and *Cryptococcus* infections [38,41].

Two points of care lateral flow assays (LFAs) are now available for IA diagnoses using serum and BAL fluid; these are (i) AspLFD (Olm Diagnostics, Braintree, UK), which detects an extracellular glycoprotein antigen [42,43], and (ii) IMMY sōna *Aspergillus* GM lateral flow assay (IMMY, Norman, OK, USA) to detect GM [44]. These are fast, effective alternatives to the Platelia *Aspergillus* Ag test, particularly where sample throughput is low [45]. Both LFA formats are sensitive and specific for haematology patients with pulmonary aspergillosis [45,46]. The LFA cross-reacts with *Scedosporium*, *Fusarium*, *Geotrichum*, and *Candida* spp. [47] whilst the AspLFD cross-reacts against *Paecilomyces* and *Penicillium* spp. [43].

The detection of the panfungal antigen, (1,3)-β-d-glucan (BDG), in the serum of patients is another approach to assist diagnosis [48]. Serum BDG is useful for the detection of many IFDs except for mucormycosis and cryptococcosis, but including PCP when interpreted with clinical/radiological signs and other microbiological markers [48,49,50,51,52]. Its performance is improved when two consecutive positive assays define a true “positive” result [16]. Although not approved by the manufacturer, the BDG testing of CSF was found to be useful in the diagnosis and monitoring of clinical response in the 2012 outbreak of *E. rostratum* meningitis [53]. 

### 2.5. Fungal Identification

Most clinical laboratories utilize a combination of morphological identification, matrix-assisted laser desorption ionization time of flight mass spectrometry (MALDI-TOF MS), and DNA sequencing to identify cultured fungi. Species identification of moulds using phenotypic methods is not possible if the organism fails to sporulate [2]. MALDI-TOF MS has revolutionized the rapid and accurate identification of both yeasts and moulds, although the success of the identification of moulds [54,55,56,57,58] relies on enhanced in-house databases [57,59].

For outbreak investigations, precise identification is essential, and comparative sequence analysis with dedicated databases, e.g., Mycobank (https://www.mycobank.org/Pairwise_alignment, accessed on 7 July 2023), is the gold standard. The primary and secondary DNA barcodes for fungal identification are the internal transcribed spacer (ITS) regions of the nuclear ribosomal RNA (rRNA) gene and translational elongation factor 1α (TEF1α) gene, respectively [60,61,62,63,64], with gene targets including the 28S rDNA D1/D2 regions, beta tubulin II, calmodulin, and RNA polymerase II subunit (RPB2) genes also used [63,65]. ITS sequencing was essential for the identification/detection of a nosocomial outbreak of *Acremonium kiliense* fungemia (see mould epidemiology) [66].

### 2.6. Molecular Typing (Genotyping)

Fungal strain typing to assess the genetic relationship of isolates is an essential component of outbreak investigation to understand epidemiological relationships between clinical isolates, or between clinical and environmental isolates, to assist with the identification of the source of the outbreak and to track and limit the spread of nosocomial infections. Ideally, the genotyping method must reproducibly assign an unambiguous result to each isolate and discriminate epidemiologically linked isolates from those that are unrelated [67]. Earlier methods such as PCR fingerprinting, random amplification of polymorphic DNA (RAPD), amplified fragment length polymorphism (AFLP), and restriction fragment length polymorphism (RFLP) analysis are subject to laboratory reproducibility and are superseded by more discriminatory methods such as microsatellite analysis, multilocus sequence typing (MLST) [68] and, today, whole genome sequencing (WGS). 

MLST uses the DNA sequencing of housekeeping genes (≥4 genes) to identify polymorphic nucleotide sites from which the population structure between isolates can be established. This method allows for database development for unambiguous interlaboratory comparisons. MLST typing schemes are publicly available at https://mlst.mycologylab.org/ (accessed on 9 July 2023), e.g., for *Scedosporium* spp. [69,70], *P. jirovecii* [30,71], and *Bipolaris* spp., and are the preferred standard for genotyping *P. jirovecii* as it can be performed directly from clinical specimens, even from samples with low fungal loads. The method is discriminatory, and an epidemic can be excluded if different genotypes are observed [14]. A drawback of MLST genotyping for *P. jirovecii* is that a single patient can be co-infected with two or more genotypes, and PCR amplification creates an artificial diversity of genotypes when applied to mixed templates [14].

Microsatellite typing is also simple, cheap, and reproducible, involving the amplification of short tandem repeat (STR) sequences. It can easily detect multiple genotypes in samples and has been used for typing *A. fumigatus*, *P. jirovecii*, and *Candida* species including *C. auris* [72,73,74,75]. Gits-Muselli (2015) demonstrated that a single *P. jirovecii* genotype may have been transmitted between 10 patients in one hospital [75]. In another study of *C. auris* comparing five typing tools (microsatellite typing, ITS sequencing, AFLP, MALDI- TOF MS, and Fourier-transform infrared spectroscopy) [73], only microsatellite typing grouped the isolates into four clusters, corresponding to the four known WGS clades at the time (Table 1).

#### Next-Generation Sequencing

Next-generation sequencing (NGS) or WGS tools are well-established preferred methods, superseding less discriminatory methods for linking genetic relatedness of bacteria and viruses. Logically, the position of WGS in mycological surveillance and tracking of outbreaks should be similar, but its implementation into routine workflows has been hindered by a number of factors. These are: (i) the substantively larger genomes of fungi (>35 Mbp for mould pathogens), (ii) the presence of ploidy, (iii) the likelihood of genome duplication and recombination events, (iv) the absence of well-developed standardized bioinformatic pipelines, and (v) the lack of comparative genome databases of an adequate breadth and depth [96,97,98]. Further, characteristics that lend themselves to constituting a reliable “reference” genome for many fungal species are not well documented, and standard reference genomes are not available, as they are for bacteria and viruses. With mould pathogens, the genome variability of those within the hospital environment and in the community needs to be understood, as this is fundamental to contextualizing the relatedness of strains in a phylogenetic investigation. Nonetheless, as experience grows and limitations including costs are addressed, the contribution of WGS in fungal outbreak investigations is increasingly recognized [99,100,101,102], with major examples summarized in Table 1 and Table 2.

## 3. Causative Pathogens and Epidemiology

### 3.1. Yeasts

Yeasts have, until recently, been considered uncommon causes of nosocomial outbreaks. Prior to DNA sequencing, conventional methods of species identification were insensitive to detecting new species and strains. Further, investigations of potential outbreaks relied on older, less discriminatory genotyping methods or only on clinical epidemiological data. This all changed with the incursion of *C. auris* into multiple regions when it was recognized that this modern *Candida* species was capable of rapid patient-to-patient transmission, could colonize and persist in the hospital environment, and could cause invasive infection and be associated with antifungal drug resistance [89,92]. This section covers the main epidemiological features of healthcare-associated nosocomial outbreaks caused by *Candida* spp. and other uncommon or rare non-*Candida* yeasts. 

#### 3.1.1. *Candida auris*

Originally misidentified as “*Candida haemolunii*” or as various other *Candida* spp. [136,137], the first recognition of *C. auris* as a pathogen was in 2009 in Japan in a patient with discharge from the ear canal [138]. Following this, it was reported as the causative pathogen of 15 cases of chronic otitis media in Korea [139] and then fungemia [140], with the earliest bloodstream isolate found to be from 1996 in Korea [140]. Yet, it was not until much later that *C. auris* infections were reported elsewhere in the Americas in 2012 (Venezuela) and in the USA and India (2013) [76,141,142]. Its emergence in Europe followed, with epidemiologic links to India suggesting its introduction from Asia [99]. Once these footholds were established, a degree of endemicity was established. WGS-enabled approaches have revealed that there were initially four, then five, distinct geographic clades of the pathogen, clearly separated from one another and each with limited genetic diversity within the clade, although delineation of sub-clades is described: South Asian (clade I), East Asian (clade II), South African (clade III), South American (clade IV), and Iranian clade (clade V) [88,143,144]. Very recently, a likely sixth clade has been identified in three non-epidemiologically linked isolates from Singapore and one from Bangladesh, where these isolates are closely related and differ by >36,000 single-nucleotide polymorphisms (SNPs) from existing *C. auris* isolates [145]. The first global genomic analyses of *C. auris* isolates indicate near-simultaneous emergence of the pathogen [144], and numerous studies have described its genetic diversity and population structure [143,144,146,147] (summarized in Forsberg et al. [141]). 

What is intriguing about *C. auris* is its potential for patient-to-patient transmission [77,92,148] and prolonged environmental persistence on dry and moist surfaces [20,137,149]. The reasons for this may include the ability of *C. auris* to form cellular aggregates and biofilm and its tolerance to high salinity and temperatures up to 42 °C [137,150]. *C. auris* is capable of colonizing the gastrointestinal (GI) tract and skin surfaces of patients, and the pathogen may be cultured from rectal swab/faecal specimens and axilla and groin skin swabs [151]. Risk factors for *C. auris* colonization/infection are similar to those of other *Candida* spp. and include prolonged time of hospital stay, indwelling central venous access devices (CVADs) and urinary catheters, and high-acuity care requirement, mechanical ventilation in particular [144,152,153]. It seems particularly suited to the intensive care unit (ICU) environment and ventilator units [92,154] and has shut down entire ICU wards due to transmission [92]. The duration of colonization with *C. auris* remains unclear; one study revealed a median duration of 8.6 months [155], but more work is needed in this area, particularly as screening is not 100% sensitive, with the tendency to repeatedly detect *C. auris* on subsequent testing despite a single negative intervening result [151]. The colonization of lanyards has been reported [156], and axillary temperature probes have been implicated as modes of transmission [77,95,142]. Table 1 summarizes the major nosocomial *C. auris* outbreaks where molecular methods have contributed to their establishment as a cluster, according to genotyping method and chronological order. For brevity, we have excluded the numerous studies that have not incorporated genotypic methods to support the outbreak [157,158].

The first reported healthcare-associated outbreaks in the USA and Europe occurred in 2013 [76,141,142], with an increasing number of reports globally since then (Table 1). Patients receiving care in ICUs—including neurosciences, burn, and cardiothoracic ICUs—appear to be at particular risk, but outbreaks have occurred in all ward types; affected individuals have mainly been adults. Outbreaks have comprised patients with invasive disease as well as those colonized by *C. auris*. Outbreak size has ranged from involving only 2 to more than 180 patients (Table 1), and there are two reports describing person-to-person transmission in COVID-19 patients [82,157]. 

One of the earliest reports involved the Royal Brampton Hospital in the U.K. in 2016, which encompassed 50 nosocomial *C. auris* cases over 16 months in a mixed surgical-medical ICU [92]. Environmental sampling of the area surrounding colonized patients demonstrated *C. auris* on the floor, trollies, radiators, windowsills, and equipment. Prospective surveillance was introduced and managed with strict infection control procedures (see below). The high degree of genetic relatedness of 15 outbreak isolates by AFLP analysis (Table 1) suggested the introduction of the infecting genotype into the hospital. 

Another study of note described an outbreak in a U.K. neurosciences ICU where after suspicion of the cluster, an intensive patient and environmental screening and intervention program, assisted by WGS studies, found that transmission of *C. auris* was linked to reusable skin-surface temperature probes [77]. All “outbreak” sequences, including those from isolates recovered from the reusable probes, formed a single genetic cluster within clade III separated by <30 SNPs. Only after removal of the temperature probes was the cluster controlled. 

In parallel with the growing number of clinical cases of *C. auris* worldwide, it is unsurprising that *C. auris* infections have emerged in hospitalized COVID-19 patients (https://www.cdc.gov/fungal/candida-auris/tracking-c-auris.html accessed on 10 July 2023). Hinrichs et al. documented the transmission between two patients in their COVID-19 ICU [157]. In another study in Miami during a local surge of patients in the COVID-19 ICU, 15 cases of *C. auris* infection were identified within concurrent admission time frames in 2020: 12 were cared for in the COVID-19 ward, and 3 were non-COVID-19 patients in a separate ward. WGS of the 15 isolates showed that the 12 spatio-temporally linked COVID-19 patients were genetically clustered with the separation of isolates ≤ 5 SNPs, and they were also closely related to 3 non-COVID patients, suggesting transmission [82] (Table 1). 

##### Molecular Genotyping of *C. auris*

A number of molecular genotyping methods have been utilized to determine relatedness between *C. auris* isolates and to support the case for a cluster or outbreak. Amongst these, AFLP analysis has been used in at least three studies [91,92,93] (Table 1). Using similar protocols [146], each demonstrated that the outbreak isolates (n = 18, 15, and 58 isolates, respectively) were closely related (85–96% genetic similarity) and were considered clonal with a clear distinction from isolates not linked to the outbreak. Techniques such as microsatellite typing and STR typing were also used [94,95], with overall good discrimination (Table 1). The most recent studies have used WGS to investigate genetic relationships and have set the premise that in general, about ≤15 SNPs separate isolates (range <2–<30) are linked in outbreaks (Table 1). 

#### 3.1.2. *Candida parapsilosis*

Prior to the meteoric rise of *C. auris* as a nosocomial pathogen, *C. parapsilosis* was the most common *Candida* species reported to cause hospital outbreaks [110,128]. Outbreaks often centred around ICUs and neonatal ICUs (NICUs) [118,124,128], with reports of fungemia triggering investigation (see Table 2). As this species produces biofilm, it is unsurprising that it has been recovered from devices such as NICU incubators and humidifiers, patient beds, nurse workstations, computers, floors, and hands of healthcare workers [113,159,160]. However, in many of the outbreaks, a point source was not identified [112].

Major outbreaks of *C. parapsilosis* which employed molecular testing are summarized in Table 2, and they have evolved from electrophoretic karyotyping to the more discriminatory microsatellite typing, which is able to identify clusters and sub-clusters, including drug-resistant clones. However, RAPD, RFLP, and electrophoretic analyses often yielded results demonstrating genetic identity reflecting their lesser discriminatory power. Thus far, only one published study has utilized WGS to investigate a possible outbreak of 31 isolates in 31 patients in a Spanish NICU, in addition to microsatellite typing [103]. Microsatellite typing was able to identify 3 clusters of *C. parapsilosis* amongst 16 isolates corresponding to epidemiologically linked cases, and these were confirmed through WGS, although the latter suggested 2 sub-populations of isolates within 1 of the main clusters, demonstrating the heightened granularity gleaned from WGS. SNP differences within clusters varied, ranging from <10 to >200 [103]. Transmission was halted after implementation of a catheter care campaign. 

Similar to *C. auris*, there have been descriptions of antifungal drug resistance emerging in *C. parapsilosis* and *ERG11* gene mutations which confer fluconazole resistance, particularly the Y132F mutation [110,111,112,118]. Given that only a small proportion of patients who have fluconazole-resistant *C. parapsilosis* have been exposed to azoles, it appears that this emergence may be due to as yet undetected transmission between patients in the hospital or the community, as confirmed by microsatellite typing [110,111,112,115,118] (Table 2). Further, there is some evidence to suggest that azole-resistant isolates are more likely to spread and be associated with invasive infection and increased mortality [110,112]. Hence, vigilance for *C. parapsilosis* infection and the need for antifungal susceptibility testing are essential. 

#### 3.1.3. *Candida tropicalis*

*Candida tropicalis* causes hospital outbreaks less commonly than *C. auris* and *C. parapsilosis*; however, when described, outbreaks have similar characteristics [132]. Specifically, the contamination of hospital environments including haemodialysis machines and ICU environments [132,133] are seen, and risk factors for invasive infection include the ICU and NICU environment, presence of CVADs, and total parenteral nutrition [132]. The organism has also been isolated from the hands of healthcare workers [132]. 

Molecular typing has been used less frequently in *C. tropicalis* outbreaks, and methods employed have tended to be of the “older generation”, including pulse field gel electrophoresis (PFGE), RFLP, and RAPD (Table 2) [131,132,133,134,135]. One study of 20 isolates from seven patients in three affiliated US haemodialysis centres found that there was an association between patient isolates and epidemiologically linked haemodialysis machines, thereby ruling out patient-to-patient transmission [133]. Another study employing computer-aided RFLP found genetically identical isolates in eight patients with sternal wound infections and a particular scrub nurse, highlighting a likely point source [135]. 

#### 3.1.4. Other Candida Species and Uncommon Yeasts

Other more uncommon *Candida* spp. and non-*Candida* yeasts have also caused nosocomial outbreaks, including *Wickerhamomyces anomalus* (formerly *Candida pelliculosa*) [161], *Candida blankii* [162], *Dilutina rugosa* (formerly *Candida rugosa*) [4], *Cyberlindnera fabianii* (formerly *Candida fabianii*) [163], and *Kurtzmaniella quercitrusa* (formerly *Candida quercitrusa*) [164]. Generally, these outbreaks were clusters of fungemia cases in ICU/NICU and were managed with standard outbreak control measures (see Section 4). 

##### *Wickerhamomyces anomalus* 

*W. anomalus* (formerly *C. pelliculosa* and *Pichia anomala*) has caused infrequent nosocomial outbreaks [161,165,166,167,168], which present typically as fungemia. Low-birth-weight infants in NICUs, premature infants, and hospitalized children appear to be at particular risk [165,168]. However, adult ICU patients are also affected [167]. Other risk factors are similar to other *Candida* infections [169]. 

In an earlier study (late 1990s) in India, an outbreak of 379 *W. anomalus* cases occurred over 23 months in a paediatric ward [165]. The use of multilocus enzyme electrophoresis (MLEE) on 40 isolates showed that strains were identical, but no external source was found. The outbreak was terminated by imposing strict hand washing. Kalkanci et al., using RAPD analysis to study four isolates from each of four infants with *W. anomalus* fungemia in the ICU, reported identical RAPD fingerprints using five different primer sets [170]. A similar outbreak of 17 fungemia cases over 18 months in a Brazilian paediatric ICU linked the placement of CVADs to fungemia, but no source was found [168]. Also using RAPD, the investigators identified a single genotype. Similar results were reported in a cluster of 10 fungemia episodes in six infants in NICU in China over 10 months, caused by a single RAPD genotype [171]. Of note, Spruitenburg et al. recently applied short tandem repeat (STR) typing to *W. anomalus* using six STR makers [172]. On analyzing 90 isolates, they uncovered 4 outbreak clusters that had simultaneously occurred across multiple units within the same hospital. When compared with WGS for 11 isolates, SNP calling identified genetic relationships highly concordant with STR typing. 

##### *Cyberlindnera fabianii* 

Nosocomial case clusters of *C. fabianni*, previously named *Hansenula* (or *Pichia* or *Candida*) *fabianii,* have been studied [163,173]. Sequencing of the ITS-1 gene region of 10 strains from 10 babies in Kuwait identified 100% sequence similarity for all [163]. WGS was utilized in another study to analyze isolates from three cases within a 2-week period in the urology department in a hospital in China [173]; the results did not support patient-to-patient transmission—two of three isolates were separated by a 192 bp SNP difference whilst the third was separated from the other two by >26,000 SNPs, suggesting different lineages [173]. 

##### *Magnusiomyces clavatus* 

This uncommon yeast (formerly *Saprochaete clavata* and *Geotrichum clavatum*) has been linked to two case clusters both occurring in haematology wards in France [174,175], suggesting either human-to-human transmission or the introduction of the organism from the environment. The first study was important in that it provided the (i) assembly of a “reference” *M. clavatus* genome and (ii) premise by which two distinct WGS-enabled clades (clades A and B) of *M. clavatus* were established [175]; a clone associated with the outbreak was uncovered. Of 18 outbreak isolates (recovered within an 8-week period), 16 belonged to clade A and differed from others by ≤4 SNPs, whilst control isolates belonged to clade B and were genetically distant [175]. No environmental source was identified. 

In a later study, Menu et al. [174] used WGS analysis to examine isolates from nine patients in a haematology ward over a 22-month period, and 10 isolates recovered from the environment, including from kitchen appliances. WGS analyses showed that all clinical and environmental isolates belonged to the same phylogenetic clade (clade “C”) and were genetically distant to both clade A and clade B from the previous *M. clavatus* outbreak [175]. Further, a dishwasher with a deficient heating system was identified as a vector of contamination; the replacement of this appliance resulted in cessation of the outbreak [174]. A more recent study [176] of an outbreak of seven *M. clavatus* fungemia in haematology patients also utilized WGS; by aligning with the reference genome of Vaux et al. [175], all seven clustered together within a single clade separated clearly from a control group. However, a comparison with results from the two French studies above was not undertaken [176]. Other hospital outbreaks of *M. clavatus* infections have been described but were not analyzed using WGS, although one study showed through MALDI-TOF MS that the main spectrum profiles (MSPs) of seven patient outbreak isolates clustered together [177]. 

##### *Rhodotorula mucilaginosa* 

*Rhodotorula mucilaginosa* is another yeast that is ubiquitous in the environment, including in hospitals, and with a strong affinity for plastics such as that in CVADs. A single-case cluster was reported by Huang et al. of *R. mucilaginosa* infection across four hospitals in China, although this was uncovered only through a retrospective examination of isolates over 10 years [178]; hence its impact went unrecognized. Microsatellite typing showed the presence of an epidemic cluster encompassing 35 strains isolated in the hospital over eight years. WGS analysis showed that 17 of 35 strains demonstrated >99% genetic similarity. Other clinical strains were divergent (40–98% single nucleotide variant similarity) [178]. 

#### 3.1.5. Clinical Consequences of Candida and Related Yeast Nosocomial Outbreaks

Candidemia of any type is associated with significant morbidity and mortality [165,179]. Mortality from *C. parapsilosis* and *C. tropicalis* candidemia can be high, particularly in vulnerable patient groups, such as NICU outbreaks [103,128]. Mortality from *C. auris* was initially reported to be high at 30–60% [91,93,140,152]. However, more recent studies have suggested lower attributable mortality [77,92]. This may be due to the earlier clinical and microbiological recognition of infection and better knowledge of appropriate treatment of this pathogen. Nonetheless, due to the significant morbidity and healthcare resource utilization associated with these infections, measures to control outbreaks are encouraged. 

In summary, WGS has been employed primarily in *C. auris* and *M. clavatus* spp. outbreaks and has shown that close genetic relatedness can be suggested by varying thresholds of SNPs depending on the species, but isolates are often not genetically identical (Table 1). For instance, with *C. auris*, <15 SNPs would be suggestive of a spread. Older molecular methods remain in use for other *Candida* species such as *C. tropicalis* and *C. parapsilosis*, which may be due to a lack of familiarity and precedent to use WGS with these pathogens. With time, it is likely that WGS will be employed more widely.

#### 3.1.6. *Cryptococcus* Species

There have been numerous reports of the utility of WGS in the investigation of genomic diversity and population studies of both *Cryptococcus neoformans* and *Cryptococcus gattii*, with clear delineation of reference genomes and the establishment of WGS findings with genetic cryptococcal genotypes established through MLST. However, these have mainly been studied in the context of sporadic infection or community outbreaks of *C. gattii* [180,181,182,183]. There have been rare reports of the nosocomial transmission of *Cryptococcus* spp. [184,185,186] and occasional reports of needlestick/laboratory cutaneous inoculation [187]. Donor-derived infections in solid-organ transplant recipients have also been rarely described [188,189]. However, in retrospect, the donors appeared to already have pulmonary parenchymal changes which could have reflected undiagnosed cryptococcosis. Wang et al. reported possible nosocomial transmission between two patients where RAPD and karyotyping showed the isolates to be indistinguishable [186], whilst in three studies employing MLST [190,191] and WGS [184], genetic analyses of isolates in the respective putative clusters were dissimilar, and in the case of a WGS-enabled study, the isolates belonged to well-separated subclades [184]. This is not surprising given the abundance of *Cryptococcus* in the environment, and where hospital pseudo-outbreaks have been reviewed, it has ultimately been found that the *Cryptococcus* is genetically unrelated [190]. 

#### 3.1.7. Dimorphic Fungi

Whilst there are well-described environmental, occupational, and geographical risk factors for exposure to dimorphic fungi which have resulted in outbreaks of infection, there are few reports of nosocomial outbreaks [192,193,194], with none reported for histoplasmosis, blastomycosis, or emergomycosis. [193]. One described cluster of nosocomial coccidioidomycosis has been linked to donor-derived infection in organ transplant recipients [195], confirmed by a WGS analysis of isolates from three organ recipients from a single donor who resided in an area endemic for *Coccidioides immitis*. The isolates from the three patients were nearly genetically identical (3 SNPs separating them). Of note, previously, only microsatellite-based methods have proven useful for molecular epidemiologic studies of *Coccidioides* spp., providing adequate separation across geographically diverse samples whilst capable of identifying genotypically identical isolates recovered from the same patient [196]. However, microsatellite methods can be biased in that they may fail to detect genomic changes outside these loci. 

#### 3.1.8. *Pneumocystis jirovecii*

*P. jirovecii* has no environmental reservoir, and historically, there have been controversies in the description of its epidemiology with uncommon reports of human-to-human transmission of *P. jirovecii* [197,198,199,200], including where healthcare workers have tested positive through *Pneumocystis* PCR post exposure to infected patients [201]. Further, *P. jirovecii* has been isolated in air samples surrounding infected patients with a gradient of higher counts as the proximity to the patient increases [202,203], providing further plausibility of human-to human transmission. The clinical context is also complicated by the fact that patients can be colonized by *P. jirovecii* without developing an infection [204]. Nonetheless, horizontal transmission has been observed, including in numerous hospital outbreaks (summarized in Delliere [198]). Probable human infectious reservoirs would be children with a primary infection, pregnant women, and patients with immunodeficiency.

The late 1990s and early 2000s saw increasing reports of nosocomial outbreaks of *Pneumocystis* pneumonia (PCP), with likely inter-patient transmission supported by more discriminatory genotyping methods [205]. These outbreaks have been almost exclusively described in solid-organ transplant recipients, and then mostly in renal transplant recipients [30,206,207,208] but also in heart [32] and liver transplant recipients [209,210,211]. Clusters in patients with haematological malignancies appear uncommon [212], and there is only a single putative cluster of *P. jirovecii* infection among patients with rheumatoid arthritis [213]. 

Case control studies supported with a systematic review [214] have indicated that risk factors for PCP among renal transplant recipients include: (a) frequent inpatient contact, (b) lack of adherence to isolation precautions, (c) first year post transplantation without chemoprophylaxis, (d) cytomegalovirus infection, and (e) age (a mean of 48 vs. 36 years old) when compared to transplant recipients who did not develop PCP. The time interval between the first and last cases of reported clusters have ranged from 2 weeks to 52 months, with the number of clustered cases varying from 3 to 83. The role of colonized patients as a potential source of *P. jirovecii* in the nosocomial acquisition of the fungus has been acknowledged by molecular studies [30,31,199].

Outbreak characterization has been furnished through methods including RFLP, MLST, and now WGS. Numerous markers have been evaluated in MLST schemes for *P. jirovecii* (ITS region, mitochondrial large subunit [*mt26S*], β-tubulin, superoxide dismutase [SOD], cytochrome b, 26*S* rRNA gene, as well as dihydropteroate synthase [*DHPS*] and dihydrofolate reductase [*DHFR*] genes), and different combinations of loci have been used in outbreak investigations and confirmed the presence of identical genotypes recovered from different patients during outbreaks [30,31,209,211,215,216]. A specific web page (https://mlst.mycologylab.org/page/PJPasic2020 [accessed on 11 June 2023]) has enabled inter-laboratory comparisons of results. In 2013, upon the evaluation of different combinations of loci, an optimized MLST scheme restricted to the three most discriminant markers (ITS, mt26S, and cytochrome b oxidase [CYB]), which achieved a discriminatory power of 0.996, was proposed [217]. One early study of an outbreak in a renal transplant unit used four-locus MLST to link 9 of 11 cases of PCP, and contact tracing found colocalization of the cases in an outpatient waiting area [30]. Another MLST study confirmed interhuman transmission in all colonized and infected PCP cases in heart transplant recipients [32].

Finally, an outbreak investigation study using MLST followed by WGS was employed to address the limitations of MLST for the detection of mixed bases at SNP positions; this was resolved using amplicon analysis with WGS [218]. The same approach was used by Charpentier et al. to investigate an outbreak in a French tertiary hospital [219].

### 3.2. Moulds

The following section focuses on nosocomial outbreaks of invasive mould infections. Generally, outbreaks of moulds tend to be related to airborne spread, local inoculation, or contact with contaminated materials, which are explored below. 

#### 3.2.1. *Aspergillus* Species

Hospital-acquired infections due to *Aspergillus* species occur more frequently when building construction or renovation are taking place or have just been completed [220,221]. These activities cause dust contamination and disperse large amounts of fungal spores, with construction activity reported as an independent risk factor for IFD. A number of outbreaks of invasive aspergillosis (IA) have been documented in healthcare settings from 1976 to 2022 [220,221], with the number of infected individuals ranging from 3 to 145 and the most common affected population being haematology-oncology patients [222]. Fungal spore transmission via the airborne route is typical, with skin and soft tissue infections less frequent. *Aspergillus fumigatus* followed by *Aspergillus flavus* have been the most frequently encountered species; however, outbreaks can result from any *Aspergillus* species or from >1 species. 

##### Risks for Nosocomial Aspergillosis-Incipient *Aspergillus* in Air

Air sampling studies, in the context of hospital-building construction works, have repeatedly demonstrated high *Aspergillus* spore concentrations, exposing those at risk. In one study, air sampling demonstrated a relatively high *Aspergillus* concentration (10 CFU/m^3^) in the corridor between two haematology wards, cumulating in several cases of IA in patients with acute leukaemia [223]. Similarly, in an antiquated haematology unit which documented 36 cases of nosocomial IA, the incidence density in pre-construction was 3.18 per 1000 days at risk versus 9.88 during construction [224]. *Aspergillus* concentration in air was 6.77 CFU/m^3^ during construction. In another cluster, contaminated aerosols from a ward vacuum cleaner were recorded, where *A. fumigatus* air concentrations of 65 CFU/m^3^ were detected [225]. It is unclear, however, what degree of air contamination correlates most with the risk of IA or whether it can, indeed, predict risk [226,227]. 

The presence (or absence) of high-efficiency particulate air (HEPA) filters also appears to be key in the risk of hospital-acquired IA. In an Australian hospital, a cluster of six cases occurred in an exposed regional haematology unit without HEPA filtration [228]. Similar case clusters have occurred in the event of disrupted airflow patterns within hospitals, in addition to the absence of HEPA filtration [229,230,231]. It is important to note that HEPA-filtration will not reduce the risk of IA entirely, with one study demonstrating that bioaerosol contamination with *Aspergillus* species was significantly increased in HEPA-filtered rooms one hour after cleaning [232], suggesting that the cleaning and maintenance of these filters is potentially a time of heightened risk for fungal aerosolization.

##### Potential Risks for *Aspergillus* Exposure from Water Environments including Biofilms

*Aspergillus* species have been isolated from potable and non-potable water sources [233]. Healthcare facilities receive water from local sources, and there is little understood about the fungal ecology dynamics prior to delivery, including during the water treatment process [234]. Fungal infection may result from direct exposure, generation of bioaerosols (inhalation), ingestion, or direct contact with skin or mucous membranes. Special sources of contaminated water exposure in the healthcare setting with source-outbreak potential include that from haemodialysis and shared shower facilities [235,236]. Moreover, *Aspergillus* biofilms have been detected in hospital plumbing and water distribution systems [234]. Temperatures of around 37 degrees favour *Aspergillus* biofilm formation in vitro, and there has been a report of seasonal variation with regard to the optimal growth of *Aspergillus* in water-favouring ambient temperatures around 20 degrees [237]. To prevent healthcare exposure to contaminated water or fungal biofilms, there have been a number of risk mitigation strategies proposed, including ultraviolet radiation, chemical pre-treatment, or thermal shock within the water pipes [238]. Despite the presence of this potential risk in the hospital environment, there have been no nosocomial outbreaks related to *Aspergillus* species where a water source has been identified. In the setting of a suspected outbreak, there is little evidence to recommend water to be routinely examined as a source (see below).

##### Molecular Methods Employed in *Aspergillus* Outbreaks

Different molecular typing methods have been used to complement the epidemiological investigation of outbreaks of IA [239] to confirm a suspected epidemiological link or exposure towards a probable source [101]. However, there are a number of limitations to this approach: firstly, environmental transmission (e.g., from construction sites), if this is to occur, is often associated with multiple strains and even species [240]. Secondly, comparative genomic studies using various methods of *A. fumigatus* have demonstrated great genetic diversity within the same patient [241]. Thirdly, many current genotyping methods are slow and costly, thereby diminishing their impact on investigation outcomes. The different methods used in the clinical setting have included STR, MLST, RAPD, sequence-specific DNA polymorphism, microsatellite polymorphism, and MLEE [239]. A study comparing the discriminatory power of these approaches among 52 clinical *A. fumigatus* isolates determined that STR typing was most suitable for use in outbreak investigation [239]. Several small cohort studies utilizing these various techniques, most commonly in a post hoc analysis, have demonstrated clonal spread with similar genotypes identified in a proportion of clinical and environmental isolates [240,242,243,244]. Equally, other studies have shown no such pattern [245]. More recently, WGS approaches have been applied to *Aspergillus* species isolated from clinical specimens, although not yet to epidemiologically link these with environmental samples [241]. No study to date has demonstrated a clear real-time advantage of using genotyping methods in the setting of a suspected nosocomial outbreak. However, where a point source is suspected (e.g., medication, device), molecular typing and WGS may have a role in focusing the search on the outbreak [101]. 

#### 3.2.2. *Scedosporium* and *Lomentopora* Species 

Invasive infections due to *Scedosporium* and *Lomentospora* species are much-feared due to the extremely limited treatment options and high fatality rates, especially in immunocompromised hosts There is a preponderance of these infections in Australia and Spain [246,247]. Table 3 highlights published studies of outbreaks caused by non-Aspergillus moulds, including *Scedosporium* and *Lomentopora* species, where molecular typing methods have been employed to investigate the outbreak.

Nosocomial outbreaks caused by these fungi appear to be uncommon. One outbreak of *Lomentospora prolificans* infection occurred among six inpatients, with acute leukaemia resulting in six deaths [248]. All patients were neutropenic and were being treated in rooms without HEPA filtration or laminar-airflow systems. No building works were coincident. Clinical and environmental samples which grew *L. prolificans* underwent PCR fingerprinting which showed patterns with identical bands [248]. However, it is not known if this method of genotyping is sufficiently discriminatory. Another cluster of four cases of fatal *L. prolificans* infection occurred again in acute-leukemic patients in the context of hospital renovation over a 1-month period [249]. Although epidemiological evidence suggested a nosocomial outbreak, environmental sampling did not yield *L. prolificans*. The four clinical isolates underwent genotyping through RAPD and PCR-fingerprinting, which showed three molecular types (two patients sharing a similar strain and the remaining two patients sharing different strains), suggesting an absence of direct patient-to-patient transmission but leaving the potential for a common source [250]. The only study attempting WGS to study the genetic relationships of six *L. prolificans* isolates from four patients clustered in space and time together with two unrelated isolates yielded inconclusive results [251]. Case isolates demonstrated a high number of mutational difference (>10,000 SNPs) between patients. 

A cluster of five healthcare-associated fungal infections due to *Scedosporium boydii* occurred in cardiac surgery patients from a single hospital, which resulted in two deaths [252]. MLST was performed, which revealed an identical sequence type on all clinical isolates. No environmental isolates were analyzed (Table 3).

**Table 3:** Nosocomial outbreaks of non-*Aspergillus* moulds, typing methods employed, and results.

Country, Year Published (Ref)	Setting and Infection Site(s) where Known	Pathogen	No. Isolates/No. Patients	Typing Method(s)	Results	Outbreak Caused by Clonal Strain (Yes/No)	Source of Outbreak	Mortality
*Scedosporium* and *Lomentospora* species								
Spain 1997 [250]	Haematology/oncology, single centre	*Lomentopsora prolificans*	14/4	RAPD, PCR fingerprinting	Four outbreak isolates consisted of three molecular types with two patients sharing a similar strain	No	Unknown	100%
Spain 2001 [248]	Haematology/oncology, single centre	*L. prolificans*	6/6	PCR fingerprinting	Clinical and environmental isolates had identical M13 fingerprint patterns	Yes; but may not be adequately discriminatory	Unknown	100%
Germany 2015 [252]	Surgical patients, single centre	*Scedosporium boydii*	5/5	MLST	Identical MLST type was found in 5 patients	Yes	Unknown	50%
*Fusarium* species complex								
Brazil 2017 [253]	Paediatric haematology/oncology, single centre	*Fusarium oxysporum*	16/7	AFLP	All 7 strains from blood and catheter tips were genetically similar	Yes	Unknown	0%
South Korea 2022 [254]	Eye surgery patients, multiple centres	*F. oxysporum*	39/39	MLST	12 clinical *F. oxysporum* isolates and 2 isolates from contaminated ocular device were of the same MLST type	Yes	Ocular viscoelastic device	Not specified
*Mucorales*								
Germany 2000 and 2019 [255,256]	Haematology/oncology, single centre	*Cunninghamella bertholletiae*	4/4	Rep-PCR, RAPD, microsatellite typing	Probable epidemiological association of the cluster isolates demonstrated by microsatellite genotyping (all were clonally related)	Yes	Possible hospital construction	75%
France 2018 [257]	Burn unit, single centre, wound infection	*Mucor circinelloides f. circinelloides*	14/7	WGS	Four clades amongst outbreak isolates with each clade separated by >290,000 SNPs; isolates within each clade varied by <20,000 SNPs; seven “control” isolates also fell within these four clades	No multiple genetically diverse strains caused outbreak	Unknown; no point source	83%
Argentina 2018 [258]	Post-arthroscopic anterior cruciate ligament repair, bone infection	*Rhizopus microsporus*	3/3	RAPD and MALDI-TOF MS	All three strains fell within one cluster using both methods; three “control” strains fell into a separate cluster	Yes; however, resolution of RAPD and MALDI-TOF MS likely insufficient	Unknown	0%
Canada 2019 [259]	Heart/lung transplantation, single centre, various sites	*Rhizomucor pusillus* (n = 2); *Lichtheimia ramosa* (n = 1)	3/3	WGS	The 2 *R. pusillus* genomes differed by >5900 core SNPs.	No, and no common source	Unknown	33%
USA 2020 [260]	Solid-organ transplantation, single centre	*R. microsporus* (n = 2), *Rhizopus arrhizus* (n = 1), *Lichtheimia corymbifera* (n = 1)	4/4	WGS	4 case isolates and 68 “control” clinical and environmental isolates showed high genetic diversity overall. Pan-genome analysis showed two patient *R. microsporus* isolates were similar, but no link between cases with environmental isolates or with other “control” isolates	No support for point source or patient-to-patient transmission for *Rhizopus* infections	Unknown	Unknown
*Miscellaneous* species								
USA 2014 [261]	Patients receiving steroid injections, multiple centres	*Exserohilum rostratum*	28/19	WGS	All 28 isolates had nearly identical genomes and were separated by </= 8 SNPs	Yes	Methylprednisolone acetate medication produced by single compounding pharmacy	Not specified
Chile and Colombia 2016 [262]	Oncology, multiple centres	*Sarocladium kiliense*	25/18 (18 clinical isolates, 7 environmental isolates)	WGS	All 18 outbreak isolates were separated by <5 SNPs) as they were from 7 strains from anti-nausea medication vials	Yes	Contaminated anti-nausea medication	Not specified

Abbreviations: AFLP, amplified fragment length polymorphism; MLST, multilocus sequence typing; RAPD, random amplification of polymorphic DNA; rep-PCR, repetitive sequence-based PCR; SNPs, single-nucleotide polymorphisms; WGS, whole genome sequencing.

#### 3.2.3. *Fusarium* Species

*Fusarium* spp. are ubiquitous in nature and tend to cause infections in heavily immunocompromised patients or in the setting of traumatic inoculation in either the healthcare setting following medical procedures or in the community [263,264]. Nosocomial outbreaks, which have at times had catastrophic consequences, have been well-reported in the setting of contaminated medical products (Table 3) [265].

Outbreaks of *Fusarium* species keratitis in contact lens wearers related to contaminated lens solutions has been described [266,267,268]. Although also a community-focussed matter, some of these outbreaks have been clearly linked to direct healthcare contact (Table 3). A same-day outbreak of post-operative endophthalmitis due to *Fusarium oxysporum* species complex (FOSC) was reported in nine patients who had phacoemulsification and intraocular lens implantation [269]. The removal of the intraocular lens resulted in clinical improvement in all cases. In a hospital in Turkey, eight cases of *F. solani* species complex (FSSC) endophthalmitis occurred following cataract surgery performed on the same day [270,271]. No environmental source was identified, although contamination of the lens irrigating solution was suspected. Another nosocomial cluster of 14 cases of FOSC endophthalmitis occurring after cataract surgery arose where the viscoelastic filling material was identified as the most likely source of infection [264]. Of note, a large nationwide outbreak of *Fusarium* endophthalmitis post cataract surgery involving 156 cases (62 confirmed and 94 probable) occurred in South Korea [254]. The suspected source was ocular viscoelastic devices from a single manufacturer (Table 3). MLST analysis performed on 12 of 39 FOSC clinical isolates and 2 isolates from ocular viscoelastic devices found that they were all of the same MLST type with follow-up data on fungal endophthalmitis resulting from the use of viscoelastic material identifying a further 89 eyes culturing *Fusarium* species over the ensuing seven-month period [272]. 

Another outbreak of FSSC endophthalmitis after cataract surgery occurred in nine patients in a Turkish hospital [273]. These cases were consecutive and occurred on the same day in the same operating room. The suspected source was the same balance salt solution used for intracameral injections for different patients, but as no molecular typing was performed, the source was not proven.

Other than outbreaks of keratitis/endophthalmitis, a cluster outbreak of catheter-related fungemia due to FOSC in seven children with cancer occurred [253]. All patients developed their infection after catheter manipulation in a specially designed room, although environmental sampling did not reveal a source. All *F. oxysporum* strains isolated from blood and catheter-tips were genetically similar through AFLP fingerprinting (Table 3). Another outbreak of IFD due to FSSC in five oncology patients occurred at a hospital in Argentina [274]. All patients had severe neutropenia and two died. On environmental screening, shower- and sink-surface samples cultured *Fusarium* species. The outbreak was successfully interrupted by changing cleaning methods and through the use of a disposable water filter. 

In another study, 10 cases of invasive *Fusarium* infection in paediatric oncology patients occurred over a two-year period in a Brazilian hospital [275]. Seven of ten cases died, and most patients had severe neutropenia without a clear infection site. Environmental water samples cultured *Fusarium* species with a demonstrated interruption of the outbreak with the implementation of water filters. No genotyping was performed. An outbreak of invasive FSSC infection occurred among 16 patients with acute leukaemia [276]. With environmental sampling, indoor air and water installations were found to be contaminated with *Fusarium* species. Twelve of fourteen (75%) were alive ninety days after diagnosis. Finally, a nosocomial cluster of *Fusarium verticillioides* complex was identified in seven immunocompetent patients over one month [277]. Episodes occurred after hospital reconstruction works, but no environmental sources were found. The outbreak ceased with enhanced disinfection and the removal of hospital patients from affected rooms. 

Most recently, in early 2023, an outbreak FSSC fungal meningitis emerged, which was related to epidural anaesthesia for cosmetic procedures in two centres in Mexico [278,279]. As of 21 July 2023, there had been 12 probable cases, 10 confirmed cases, and 9 deaths [280]. 

#### 3.2.4. Mucorales

Outbreaks of IFD due to the mucormycosis described have been typically linked to contaminated healthcare supplies or other environmental sources [281,282]. Characteristics of documented hospital outbreaks caused by Mucorales were summarized by Walther and colleagues [283]. The most common route of transmission was contact with the source, and the most common causative pathogen was *Rhizopus* species. The number of cases ranged from 2 to 12, and the common sources cited where known included adhesive bandages, linen, wooden tongue depressors, and air ventilation systems. 

Suboptimal conditions of washing, drying, and storage of hospital linen resulted in six immunosuppressed patients developing pulmonary and cutaneous *Rhizopus microsporus* infections [284]. The identification of all the fungal isolates was confirmed through ITS sequencing; however, no further genotyping was performed. Further, air samples taken from the designated laundry were culture-positive for *R. microsporus*. 

Another cluster of four hospitalized patients with invasive cutaneous *Rhizopus* infections also occurred following exposure to contaminated laundry carts [285], as did a similar occurrence in five paediatric oncology patients [286]. Finally, a large contemporary US-based study which performed a fungal culture on freshly laundered linen at 15 transplant and cancer hospitals recovered Mucorales in >10% of these [287]. No genotyping was undertaken. Rickerts et al. documented a cluster of pulmonary infections due to *Cunninghamella bertholletiae* in four haematology patients [255]. Repetitive sequence-based PCR (rep-PCR) and RAPD analysis of clinical isolates performed retrospectively 10 years later [256] demonstrated clonal relatedness between all four patients (Table 3).

Due to an unprecedented rise in COVID-19-associated mucormycosis cases in India, a multicentre hospital-based environmental sampling study was undertaken and found that 11% of air-conditioning vents and the used masks of 2% of patients were colonized by Mucorales [288]. In addition, Mucorales grew from 22% of indoor and 54% of outdoor air samples. *Rhizopus* species was the most frequent pathogen. This study highlights that depending on the setting and region of the world studied, there may be a high incidence of Mucorales in the environment, and additional insults such as viral infection may place already vulnerable patients at a higher risk of Mucorales infection. 

In the three more recent studies that have harnessed WGS to confirm an outbreak, the analysis results of a relatively small number of *Mucor*, *Rhizopus*, and *Rhizomucor* isolates showed that there was a very high genetic diversity amongst the case outbreak isolates (separated by approximately 20,000 SNPs to >200,000 SNPs) (Table 3) and similar diversity amongst non-outbreak isolates and environmental isolates. Garcia-Hermoso et al. utilized WGS to answer questions regarding a possible nosocomial cluster of six patients with a *Mucor circinelloides* f. *circinelloides* wound infection [257], where they studied 21 isolates (14 outbreak and 7 unrelated isolates). This study was important as it found, unexpectedly, that the threshold of about 5296 SNPs was a cutoff below which two isolates could be designated as being the same strain. Contrary to the initial notion that a single-strain clonal transmission caused the outbreak, the cluster was due to multiple unrelated strains present in the environment. 

#### 3.2.5. *Exserohilum* Species 

A large outbreak of *E. rostratum* meningitis together with (para) spinal abscesses occurred in the USA where over 13,000 individuals were exposed to contaminated methylprednisolone acetate, and over 750 developed fungal infection, with 55 deaths [261,289,290,291]. Twenty-two *E. rostratum* isolates obtained from 19 patients were submitted for WGS, which revealed only seven non-parsimonious concordant SNPs within the set of isolates, and most outbreak isolates had identical genomes, suggesting the isolates were closely related and supporting a common source of infection (Table 3). Using an *E. rostratum* real-time PCR assay on body fluids further assisted the delineation of the extent of the outbreak by identifying a further 57 patients [29].

#### 3.2.6. *Sarocladium* Species 

*Sarocladium* spp. are environmental moulds found in soil and plant debris and rarely cause human infection. However, a nosocomial outbreak of *Sarocladium* (formerly *Acremonium) kiliense* bloodstream infection occurred due to contamination of vials containing ondansetron, which was administered to oncology patients [262,292]. This cluster comprised 67 cases which occurred across eight different hospitals. A total of 25 outbreak isolates (18 from patients and 7 from medical vials) underwent WGS, which revealed that all were nearly indistinguishable (separated by ≤5 SNPs) [262]. 

Three further cases of suspected catheter-related bloodstream infection due to *S. kiliense* in a haematopoietic stem cell transplant unit has been described [66]. Despite extensive environmental sampling, no source was identified. No genotyping was performed. 

In summary, for non-*Aspergillus* mould fungi, older less discriminatory genotyping methods and MLST still underpin many outbreak characterizations (Table 3), although the study on *Sarocladium* utilized WGS with a good resolution [262]. For WGS analyses of other genera, given the genomic diversity of Mucorales, *Lomentospora*, and *Scedosporium* species (there are few data on *Fusarium* spp.), it is necessary to first establish the diversity as it exists from multiple strains from patients and the environment during epidemiological investigations; the challenges of this practice are acknowledged. 

## 4. Management

### 4.1. Containment and Infection Control

When assessing a potential nosocomial fungal outbreak, a key issue is the lack of well-established incubation periods for fungal pathogens [293,294]. This is particularly an issue with airborne pathogens that tend to cause clinical infection in the immunocompromised, such as *Aspergillus* spp., where there is likely a sequence of exposure, followed by colonization, and then clinical disease onset and possible dissemination which may be precipitated by the immunocompromised state [294]. Along this timeline, it can be difficult to ascertain when the colonization of the patient occurred, as the definition of colonization is not clear-cut, and there could be a long period between colonization and the episode of clinical disease [294]. Murine models of *Aspergillus* infection have demonstrated neutrophil accumulation 4 days after immunosuppression, moderate hyphal proliferation and lung parenchymal invasion by 7 days, and marked hyphal proliferation and development of necrotic lesions by 10 days [295]. A study of patients with acute myeloid leukaemia who developed aplasia post chemotherapy found a median time from aplasia to proven/probable IAs of 15 days [296], providing some insights into the timeline between the onset of a profound immunocompromise state in a colonized patient and infection. The incubation period for mucormycosis is also not well established. However, some information can be gleaned from studies of discrete episodes of percutaneous inoculation in the setting of trauma. One review of 168 patients found a median incubation period of 8 days for cutaneous mucormycosis post percutaneous injury [297]. Another study post percutaneous exposure found an incubation period ranging from 6 to 24 days [298]. How this translates in the setting of pulmonary or rhino-sinoorbital disease in the immunocompromised is unclear. Regardless of the uncertainty of incubation periods, given the dire consequences of invasive mould infections, any contribution which the healthcare setting may have in a cluster of infections needs to be carefully scrutinized.

The management of a nosocomial fungal outbreak requires several initial steps. Firstly, there is a need to detect the presence of a potential outbreak, which can be challenging. Surveillance of fungal infections has traditionally been labour-intensive and time-consuming [299]. Given this, surveillance has largely been sporadic and project-based rather than routine; hence, patterns may only be detected in significant retrospect. Routine surveillance could detect patterns of concern, which would prompt investigation into any issues, be that nosocomial or external, which is of clinical importance regardless of source. Artificial intelligence and electronic surveillance are likely the future approaches, with some work already ongoing in this arena promising to provide more streamlined and reliable routine data in the future [300]. 

Once a potential outbreak is detected, this needs to be confirmed. To do this, fungal species identification, antifungal susceptibility testing, and molecular testing (as above) can aid in clarifying the relatedness of the organisms. On the confirmation of a likely outbreak, a thorough review of cases for infection identification date, the hospital environment in these settings, and host risk factors is required to identify any commonalities indicating a potential source [293,301]. The involvement of a multidisciplinary team of infection control, clinical teams, microbiology, and engineering is essential, and active surveillance for further cases is recommended [301]. 

*C. auris* and, to a lesser extent, other yeasts can behave like a typical nosocomial pathogen, with patient-to-patient, patient-to-healthcare worker, and environment-to-patient spreads [77,84,94]. Mould infections tend not to behave in this way and are more likely to either have airborne spread from aerosolization, soil/vegetation exposure with inhalation, or contamination of medical product/equipment, and consequently, the approaches to further outbreak management are different [101]. 

#### 4.1.1. *Candida auris* and Other Candida Species 

Dedicated guidelines have been produced to inform infection control practices against *C. auris*, including from the US Centers for Disease Control and Prevention (CDC), Europe, and Australia [302,303,304,305]. A detailed description of these recommendations is out of scope for this article; however, key recommendations are discussed.

##### Isolation of Infected or Colonized Individuals with *C. auris*

Patients should be situated in a single room, with an ensuite bathroom, where possible; however, cohorting is acceptable if necessary [302,304]. Standard and contact precautions should be instituted, and it is recommended to minimize the number of staff caring for these patients [302,303,304]. Single-use patient equipment should be used where possible [302,304,305]. The CDC have developed recommendations for subacute care and nursing home transmission-based precautions [306], which strike a balance between preventing transmission and practicality. Adequate hand hygiene is essential, and alcohol-based hand rubs are advised for hands not visibly soiled [302,304,305].

The duration of colonization is unclear, but prolonged colonization (up to a year) has been described, and surveillance swabs can have variable sensitivity [155,307]. Therefore, it is not recommended that patients who have been known to be infected or colonized be de-isolated during their admission [302,303,305]. The provision of patient education relating to transmission risks is important [304].

##### Who and How to Screen for *C. auris*

Composite swabs of the axilla and groin [302,308] are recommended to screen for *C. auris*. Close contacts (defined as current-room contacts and past-room contacts within the previous month) and those at a high risk of colonization are suggested to be screened in the first instance [308]. Other high-risk individuals include those transferred from endemic facilities or who have had overseas healthcare admission with at least an overnight stay in the last 12 months. Close contact and other high-risk patients can be de-isolated after three consecutive negative screens taken at least 24 h apart [302]. If there is suspected local transmission based on the screening of close contacts, a wider screening strategy is generally indicated, including the screening of ward contacts and/or a point prevalence survey of the ward [302,308].

Environmental sampling is generally not recommended; however, it may be indicated to perform investigations for an outbreak source [302,304].

##### Cleaning and Disinfection

As the environment is a reservoir for *C. auris*, cleaning is of paramount importance when managing outbreaks. Not all agents labelled as fungicidal are effective in cleaning in the setting of *C. auris* [309,310,311]. Chlorine-based disinfectants (sodium hypochlorite 0.39–0.825%) and peracetic acid (1200 ppm) with hydrogen peroxide (<1%) are generally effective [310]. Quaternary ammonium compounds are not efficacious [310,312], and their use is not recommended [302,304]. Non-touch disinfection techniques such as UV light are not recommended as the sole cleaning technique [302,304]. The frequency of cleaning of patient rooms should be at least once a day [302,304].

When a patient is discharged or relocated, terminal cleaning of the patient environment is advised, where there is thorough cleaning using 1000 ppm of available chlorine with detergent or hydrogen peroxide [302,303], with training of staff required. The thorough cleaning of reusable medical devices and equipment is also advised, with the CDC recommending an FDA-cleared liquid chemical sterilant for this purpose.

##### Other Measures

There are inadequate data to recommend the routine decolonization of patients colonized with *C. auris* [302,304]. The documentation and alerts of the patients’ *C. auris* status are essential, and communication with transferring health facilities of *C. auris* positive-patients [304,305] is strongly advised. Regular auditing and feedback of hand hygiene, personal protective equipment (PPE) donning and doffing, and environmental cleaning are suggested. 

In general, the containment and infection control principles and recommendations for *C. auris* infections will suffice for outbreaks caused by other *Candida* and yeast species. *C. parapsilosis* (but also other yeast species), in particular, is found on human skin, including the hands of healthcare workers and, on occasions, on fomites [105,130,135]. General good hygiene measures and standard infection control with regard to oral and gastrointestinal secretions should be attended to. Attention to best-practice aseptic techniques for the insertion and maintenance of invasive devices is important, particularly for CVADs. Isolation in a single room where possible is advised, otherwise cohorting of patients is acceptable. Surveillance for additional cases should be carried out (see above). 

#### 4.1.2. Mould Outbreaks—Infection Control and Management

A review of a mould outbreak, as outlined above, will potentially highlight a common factor, such as linen, food source, contaminated surgical equipment, or medical products such as injectable corticosteroids, to guide further intervention. In addition to this, an important factor that may be implicated is construction in the vicinity of a healthcare service (see above for details of prior outbreaks). The principles of cohorting, cleaning, and surveillance are similar to those of yeast outbreaks. 

##### Building Works—Prevention of Outbreaks

Hospital building works pose a risk of invasive mould infection (as above). The key to reducing risk is the prevention and planning of risk mitigation strategies prior to construction/renovation or demolition. There are several best-practice recommendations in this area [299,313,314,315,316], and a summary of principles are discussed herein. 

Infection control consultation and planning of risk reduction prior to construction are essential, and guidelines outline risk assessment matrices and recommendations based on the level of perceived risk that building works pose to patients. A thorough review of mechanical air filtration and supply to high-risk areas is required prior to works commencing [301]. Measures to reduce patients’ exposure to dust, stagnant water, and damp areas are outlined in best-practice guidelines. Regular inspections by infection control practitioners and engineers, with a monitoring checklist of essential measures, is advised; targeted serial environmental sampling can be considered as part of enhanced surveillance [301] (see below for details). 

In wards where high-risk immunosuppressed patients are cared for, such as allogeneic stem cell transplant recipients, it is recommended that patients are placed in positive-pressure rooms with HEPA filtration systems [299,301,316]. These systems also need to be adequately maintained [225]. Patients may need to be relocated if the above is not possible. Provision of education to patients about invasive mould infection and ways to reduce risk of exposure is advised, and masking up when traversing sites of high risks can be suggested, with some efficacy described in one study [317]. Antifungal prophylaxis should be employed as per best-practice guidelines [318], and it is an institution-based decision as to whether expanded antifungal prophylaxis is warranted during high-risk construction periods. 

##### Environmental Sampling 

The environmental investigation of healthcare-associated mould outbreaks is pertinent to the overall infection control response. Tools and checklists are available to aid in the systematic approach to the investigation of relevant environmental fungi such as *Aspergillus* species and Mucorales [293,319]. Environmental sampling may also be warranted and should be guided by epidemiological findings and results from other environmental assessments (e.g., inspection of the heating, ventilation, and air conditioning [HVAC] system) [319]. 

However, it should be understood clearly that results from environmental sampling are often unlikely to provide definitive conclusions, and it is important to focus on key high-risk areas that will then inform a sampling strategy. These include general elements (e.g., evidence of fungal growth, water damage, or air intrusion from outdoors), ventilation and air conditioning (e.g., damaged filters, maintenance and repair issues, airflow disturbances), facility and built environment (e.g., construction and renovation, roof leaks, efficacy of environmental cleaning), and laundry and textiles (e.g., storage, cleaning, and laundry operations) [319]. Environmental sampling to find yeasts involved in nosocomial outbreaks, namely, *C. auris*, is generally not recommended [320] (see Section 4.1.1). 

Where a decision for air sampling is undertaken, it is important to utilize only validated methods [321]. Air sampling techniques are either passive (gravitational) or active (volumetric) approaches. Passive air sampling typically involves the use of settle plates, which exposes nutrient-rich solid media to the air. Because it is dependent on gravity, there is a bias towards fungi with larger spore sizes. However, it is simple and cheap to perform and requires no additional laboratory equipment. Other important physicochemical properties of fungal conidia also play a role in the adhesion to environmental surfaces and propagation in ambient air [322]. This may affect the approach taken to environmental sampling as well as its yield. Conidia from Mucorales (e.g., *Rhizopus* species) are hydrophilic and will aggregate within liquid droplets which rapidly sediment on surfaces [323]. In contrast, conidia from *Aspergillus* species are more hydrophobic and tend to propagate in air for longer before falling onto surfaces [324,325]. Sedimentation velocity is a function of aggregation among microbial aerosols, which depends largely on the manner of spore formation by fungi and the way in which spores are released [322]. Active air sampling involves the use of a specialized device which draws in a predetermined volume of air over a defined period of time [326]. This is a quantitative method whereby specimens are examined by direct microscopy, culture, or molecular methods. Differences in collection time, sample volumes, and airflow disturbances will all affect the types of quantity of fungi captured [327]. Direct-examination (i.e., non-viable) samples can also be collected through spore traps (i.e., an inertial impactor with air sampling cassettes) [321]. Samples are examined microscopically for spores, hyphae, and other fungal structures and can usually only be identified to the genus level. Results are reported in number of spores per cubic metre of air. A similar direct, non-viable method for sampling uses a tape or slide with an adhesive which is pressed against the environmental surface of interest (e.g., Bio-Tape^TM^ system), which is then sent to the laboratory for analysis [328]. There are numerous commercial air samplers available, although there are no recognized standardized procedures for air sampling in the setting of a suspected outbreak. The laboratory processing of collected air samples should be accredited for environmental testing.

Water sampling can also be performed, as hospital water distribution systems may be a source of invasive moulds such as *Aspergillus*, *Fusarium*, and Mucorales [329,330]. However, this is time-consuming and limited by the variability in collection protocols and analyses; hence, results need to be interpreted with some caution. Water sampling would only be recommended where a water system was strongly suspected to be the source of the outbreak [301]. Standard fungal culture and molecular methods (e.g., PCR) may be used to detect culprit fungi in water-based samples contaminated with fungal spores, but the yield of such approaches should be tailored to each case.

##### Investigation of Nosocomial Mould Outbreaks with Unclear Source

In addition to the general measures outlined for the investigation and management of a potential fungal outbreak outlined above, other measures recommended in the case of a suspected nosocomial mould outbreak include consideration of the relocation of high-risk patients, increased PPE use (i.e., n95 masks), reduction of unnecessary thoroughfare to the area of concern, and sealing of patient care areas [301]. Importantly, in the setting of a mould outbreak (as opposed to yeast), the finding of an increased rate of infections from unrelated strains of mould does not rule out the possibility of a healthcare-associated outbreak, as the common factors leading to mould infection may still be a healthcare-associated issue, for instance, construction in the vicinity of the hospital site [301].

### 4.2. Antifungal Therapy

Patients colonized by fungi do not usually require treatment. However, those at very high risks of invasive infection can benefit from prophylaxis [318]. Patients with IFD require antifungal treatment, which varies with the causative pathogen, the site of infection, and, due to regional variation in drug resistance patterns, the geographic region [142,331]. Whilst detailed descriptions for the treatment of each pathogen are beyond the scope of this review, Table 4 summarizes the generally accepted first- and second-line agents from consensus guidelines for the major groups of pathogens. 

*C. auris* is known to often be resistant to fluconazole, and traditionally, echinocandins have been the empiric therapy of choice for *C. auris* infections [302]. Concerningly, descriptions of emergent resistance to echinocandins have been described in the setting of established hospital endemicity [89]. This highlights the need for health services to double their efforts in preventing nosocomial transmission, as well as the need for antifungal susceptibility testing to identify drug resistance.

## 5. Conclusions

In conclusion, nosocomial clusters caused by fungi, whilst not common, are unpredictable. Both yeast and filamentous fungi cause outbreaks, and general and specific risks for each of these should be appreciated. Early detection and confirmation (or not) of the outbreak is essential for the diagnosis and treatment of affected patients and for termination of the outbreak. Sampling of the environment, including the air in mould outbreaks, for the pathogen maybe indicated. Genotyping of epidemiologically linked isolates is strongly advised utilizing a sufficiently discriminatory method, with WGS expected to be increasingly widely used. In addition to treating affected patients with antifungal drugs and other measures such as source control, the management of the outbreak encompasses input from a multi-disciplinary team with sound epidemiological investigation and infection control measures, including screening for additional cases, patient cohorting, and strict hygiene and cleaning procedures. More automated methods of fungal infection surveillance would greatly aid in earlier detection of potential nosocomial outbreaks and should be a focus of research moving forward.

## Figures and Tables

**Table 1:** *Candida auris:* nosocomial outbreaks that have incorporated genotypic methods to investigate isolate relatedness.

Country, Year (Reference)	Setting	No. Patients/No. Outbreak Isolates Analyzed	Typing Method	Genetic Diversity	Drug-Resistance Genes and Mutations
USA 2017 [76]	Single centre New Jersey (NJ), single centre Illinois (IL)	2/2 NJ 2/2 IL	WGS	NJ: 2 isolates assigned to clade I (separated by <10 SNPs) IL: 2 isolates assigned to clade IV (separated by <10 SNPs)	Not reported
UK 2018 [77]	Neurosciences ICU (over 3 years)	70/72	WGS	All outbreak isolates formed a single genetic cluster (clade III) comprising 72 isolates from 37 patients and 6 temperature-probe isolates (separated by <30 SNPs between isolates); close matches between patient and temperature-probe samples	Not reported
Saudi Arabia 2020 [78]	Single centre, various wards	7/7	WGS	All 7 isolates assigned to clade I but 2 sub-clusters (for both clusters, isolates within separated by <10 SNPs)	Not reported
USA 2020 [79]	Surgical ICU, liver transplant	5/5	WGS	All 5 isolates assigned to clade I (separated by <16 SNPs)	Not reported
Italy 2021 [80]	COVID ICU	10/10	WGS	All 10 isolates assigned to clade I (separated by mean/median SNPs 7)	All isolates resistant to fluconazole and amphotericin B *ERG11* K143R mutation all isolates *TACB1* A640V mutation all isolates
Hong Kong 2021 [81]	Single centre	15/19	WGS	All 19 isolates assigned to clade I (separated by <13 SNPs)	Not reported
USA 2021 [82]	Single centre; COVID-19 ward, non-COVID-19 wards	15/15 12 in COVID-19 ward, 3 in non-COVID-19 ward	WGS	12 isolates with spatio-temporal link: all assigned to clade III (separated by ≤5 SNPs); 3 non-COVID ward isolates with no spatio-temporal link also closely related to COVID ward isolates (≤5 SNPs)	Not reported
USA 2021 [83]	Single institution	14/20	WGS	All 20 isolates assigned to clade III (separated by 1–9 SNPs); however, unable to distinguish 5 outbreak isolates from non-epidemiologically unliked isolates (n = 15)	Not reported
USA 2021 [84]	Multiple LTACH/vSNFs in region (9 facilities)	182/81	WGS	81 isolates assigned to clade III (separated by <11 SNPs); suggesting significant spread among these facilities in the region	Not reported
Canada 2021 [85]	ICU of community health facility	4/4	WGS	All 4 isolates assigned to clade I (separated by ≤15 SNPs)	*ERG11* Y132F mutation all isolates
UK 2022 [86]	Single hospital (ICU, cardiothoracic, cardiac medical)	16/21	WGS	18 isolates assigned to clade I (separated by mean of 4 SNP); 4 environmental isolates also belonged to clade I; 3 isolates assigned to Clade III (separated by mean 12 SNPs); 3 introductions to hospital, with subsequent spread	*ERG11 Y132F* mutation-in clade I isolates (n = 18 clinical and 4 environmental isolates), *ERG11* F126L mutation in clade III isolates (n = 3)
France 2022 [87]	Burn ICU	2/2	WGS	Both isolates assigned to clade I (separated by ≤12 SNPs)	Not reported
Iran 2022 [88]	5 isolates from Iran (different settings)	5/5	STR and WGS	New clade identified (clade V) (with isolates within separated by <100 SNPs) and separated by >200,000 SNPs from other clades	*ERG11* Y132F mutation (n = 1), *ERG11* I466L mutation (n = 1), *TAC1b* D599G mutation (n = 1)
Lebanon 2022 [83]	Single centre (ICU, resp care, CCU neuro ICU, ED)	21/28	WGS	28 isolates assigned to clade I (separated by 1–6 SNPs)	All fluconazole and amphotericin B resistant *ERG11* Y132F mutation all isolates *CDR1* D709E mutation all isolates
Italy 2023 [89]	ICU	503/60	WGS	All 60 isolates assigned to clade Ic (separated by median of 8 SNPs, IQR 5–12); in comparison, non-epidemiologically linked “control” isolates reported as less related (separated by median of 14 SNPs, IQR 12–16)	All 60 isolates resistant to fluconazole 2 isolates resistant to caspofungin Gene mutations identified in azole-resistant isolates: *CDR1* (V704L substitution), *ERG1* (K143R), *TAC1B* (A640V) *HMG1* (P238H)
Qatar 2023 [90]	9 major hospitals	65/76	WGS	All 76 isolates assigned to clade I (separated by ≤21 SNPs)	*ERG11* Y132F mutation (n = 120), K143R (n = 2) *CDR1* E709D mutation (n = 12), V704L (n = 2) *FKS1* S639F mutation (n = 2) and S639Y (n = 1) One pan-resistant isolate also harboured a premature stop codon in *ERG3* and novel mutations in *CDR2*
Venezuela 2016 [91]	ICU, single centre	18/18	AFLP	All isolates (n = 18) clustered together with overall similarity of 85% with two sub-clusters; cluster was distantly related to strain type of *C. auris*.	Not reported
UK 2016 [92]	Cardiothoracic ICU single centre	50/15	AFLP	All isolates (n = 15) formed a distinct cluster with high degree of relatedness; clearly separated from isolates (n = 48), not linked to cluster	Not reported
Spain 2018 [93]	Single centre ICU	140/58	AFLP	All isolates (n = 58) in a single cluster, with overall genetic similarity of >96%	Not reported
Kuwait 2020 [94]	HDU of secondary care hospital (long-term care)	71/71	12-locus short tandem repeat (STR) analysis	With exception of 4 isolates from a single patient which differed only at a single locus, all outbreak isolates (n = 71) and environmental isolates (n = 7) had identical STR patterns and belonged to clade Ic; control clade I isolates not linked to outbreak also differed from outbreak isolates at one locus only	*ERG11* Y123F mutation present in nearly all isolates; 4 isolates (one patient) had the *ERG11* K143R mutation.
Brazil 2021 [95]	Single centre ICU	6/6	Microsatellite analysis	All 6 isolates belonged to clade I but were of three different STRs, signifying three different strains (~85% related)	Not reported

AFLP, amplified fragment length polymorphisms; CCU, coronary care unit; ED, Emergency Department; HDU, high-dependency unit; ICU, intensive care unit; LTACH, long-term acute care hospital; neuro, neurosurgical; resp, respiratory; SNP, single-nucleotide polymorphism; STR, short tandem repeat; vSNFs, ventilator skilled nursing facilities; WGS, whole genome sequencing.

**Table 2:** Outbreaks caused by *Candida* species other than *Candida auris*: characteristics and results of genetic analyses according to genotype.

Country, Year Published (Reference)	Setting and Infection Site(s)	No. Patients/No. Isolates Studied	Typing Method(s)	Results	Nosocomial Spread Supported (Yes/No) and Drug Resistance Mutations Identified
*Candida parapsilosis*					
Spain 2021 [103]	Single centre NICU	31/31	Microsatellite typing and WGS	16 isolates belonged to 3 microsatellite clusters (15 were singletons). Supported by WGS analysis (but with varying numbers of SNP differences between and within clusters)	Yes, for 16 isolates linked to 3 clusters
Sweden 2009 [104]	Haematology ward	9/4	MLST RAPD Microsatellite typing	All isolates from cluster were of same genotypic profile through MLST and microsatellite typing	Yes, by MLST and microsatellite typing
Taiwan 2011 [105]	NICU *	14/18 + 7 HCW hand isolates	Microsatellite typing	11 different subtypes, 2 main subgroups of highly related isolates with association with HCW isolates	Results clearly implicate HCW hands, better discrimination compared to prior methods (PFGE, RFLP, RAPD)
Austria 2012 [106]	Adult cardiothoracic ICU	50/83	Automated repetitive sequence-based PCR Microsatellite typing	Automated repetitive sequence-based PCR not discriminatory: by microsatellite typing, 9 genotypes identified but 2 genotypes prominent	Yes—microsatellite typing suggested transmission of some genotypes but also suggested other isolates were not part of an ongoing outbreak
Brazil 2013 [107]	NICU	11/11	Microsatellite typing	9 isolates with same genotypic profile, 2 were distinct	Yes, for 9 isolates
China 2016 [108]	Single centre, 13 wards	144/201	Microsatellite typing	45 different genotypes, but 2 genotypes very prominent with n = 74 and n = 23 in clusters	Yes, for 2 clusters identified
Italy 2020 [109]	Single centre (5 wards)	70/70	Microsatellite typing	4 clusters identified	Yes, some nosocomial transmission identified for each cluster
Turkey 2021 [110]	Single centre (all wards)	47/58	Microsatellite typing	31 microsatellite genotypes, 2 major clusters (n = 36 and n = 22 isolates, respectively)—one fluconazole-resistant isolate and one fluconazole susceptible, and a number of fluconazole-non-susceptible isolates; 8 sub-clusters	Yes; multiple clusters identified including cluster of fluconazole-resistant isolates carrying the *ERG11* Y132F mutation or *ERG11* Y132F + G307A mutations; other drug mutations were *ERG11* G458S
Mexico 2021 [111]	Single centre (all wards)	12/12	Microsatellite typing	10 microsatellite types, 2 closely related clusters, 1 isolate was an outlier	Yes; *ERG11* I197I and Y132F mutations identified
France 2021 [112]	Single centre (all wards)	18/26 fluconazole- res isolates/18	Microsatellite typing	6 microsatellite types: 2 clusters (both fluconazole-resistant isolates) with low diversity within the clusters	Yes. *ERG11* Y132F mutation identified
Japan 2022 [113]	NICU	3/3	Microsatellite typing	3 patient isolates were identical to each other and to 2 environmental strains	Yes, with link to environmental source; drug mutations not reported
Brazil 2022 [114]	Two hospitals with shared cardiac ICU	31/31	Microsatellite typing	Similarity of >94% amongst fluconazole-resistant isolates (n = 24)	Yes—nosocomial spread of fluconazole-resistant isolates *ERG11* Y132F and *ERG11* R398I mutations
Brazil 2022 [115]	Single centre	57/60 (all isolates were fluconazole- resistant	Microsatellite typing	51 of 60 fluconazole-resistant isolates were in same cluster	Yes; *ERG11* K143R mutation; *TAC1* L518F mutation; *CDR1* overexpression; *FKS1* E1939G mutation-conferring echinocandin tolerance
Iran 2022 [116]	Paediatric ICU	42/50	AFLP Microsatellite typing	AFLP—2 main clusters; microsatellite—5 clonal lineages	No; microsatellite typing suggested clonal spread unlikely
France 2023 [117]	2 Parisian Hospitals	39 fluconazole- resistant isolates	Microsatellite typing	2 clones with minimal intra-clonal diversity	Yes, spread of 2 fluconazole-resistant clones
France 2023 [118]	Single centre ICU	17/17	Microsatellite typing	All isolates of the same clone, 14/17 near identical	Yes; suggests nosocomial spread of fluconazole-resistant isolates; *ERG11* Y132F mutation identified
Turkey 2019 [119]	ICU	13/13	Repetitive sequence-based PCR	Genetic similarity of >98% for 11 isolates; remaining 2 isolates dissimilar	Yes, for 11/13 isolates
USA 2004 [120]	Single centre- medical, surgical, neurosurgery ICU wards	15/15	RAPD	All 15 isolates: genetic similarity of >85%	Yes
Spain 2004 [121]	Paediatric ICU	16/11	RAPD Electrophoretic karyotyping	RAPD lacked discriminatory power; karyotyping + morphotyping more discriminatory	Yes, karyotyping and morphotyping helpful
Taiwan 2007 [122]	NICU *	17/23	RFLP RAPD	All blood isolates (n = 14) had high genetic homogeneity; colonising isolates (n = 9) genetically heterogeneous, however; 7/8 of HCW hand isolates same strain as blood isolates	Yes; strongly suggested clonal outbreak related to HCW hands
Turkey 2008 [123]	Neurological ICU	4/4	RAPD	All outbreak isolates had same profile	Yes
Mexico 2010 [124]	Single centre NICU, preterm neonates	3/6	RAPD	6 isolates from 3 patients fell into two RAPD patterns, (A and B), as did two isolates from healthcare workers (A and B)	Yes, suggested HCW hands as source of outbreak
USA 2008 [125]	4 separate ICU outbreaks 3 NICU 1 children’s hospital TPN program	34/34	Southern blot Cp3-13 DNA hybridization	NICU Outbreak I—100% identical NICU Outbreak II—100% identical NICU Outbreak III—2 circulating strains; TPN program—each patient had their own type causing recurrent infection	Yes, for NICUs with spread, and indicated TPN issue related to individual patients with recurrent infection rather than spread
USA 2004 [126]	Single centre	5/5	DNA fingerprinting	Genetically identical for all 5 clinical and 1 environmental isolate; 2 environmental isolates were genetically distinct	Yes, for all clinical isolates and 1 environmental isolate
USA 1996 [127]	NICU	4/6	PFGE	All 6 isolates had identical chromosomal banding	Yes
Taiwan 1999 [128]	NICU and branch hospital *	14/14	PFGE	4 genotypes identified amongst 14 isolates (4 clusters) Of 75 hand isolates, some were genetically closely related; 26 environmental samples also analyzed—all negative for *C.parapsilosis*	Yes, likely multiple introductions from hand source
USA 1997 [129]	NICU	15/19	Electrophoretic karyotyping	8 different karyotypes amongst 19 isolates; 5 isolates from 4 infants of the same karyotype but not temporally related	No, different karyotypes encountered, and those related had no epidemiologic link
Brazil 1998 [130]	Haematology/BMT/Oncology	5/6 + HCW hands (n = 3)	Electrophoretic karyotyping	2 different profiles identified, which were also identified on HCW hands	Yes, 2 clustered identified associated with HCW hands
*Candida tropicalis*					
Korea 2005 [131]	Surgical ICU- candiduria	34/34	PFGE	Identical banding for 34 patient isolates and 6 environmental strains	Yes
Serbia 2020 [132]	Single centre	2/2	PFGE	Identical banding for 2 isolates	Yes
USA 2021 [133]	3 affiliated HD centres	7/20	PFGE	4 strain types amongst 7 isolates and together with 11 environmental isolates from HD machines	Association of strain types with environmental strains; ruled out patient-to-patient transmission, ruled in environmental source
Greece 2003 [134]	Single NICU	8/14	RAPD RFLP	2 genotypes identified (7 patients with A, 1 patient with B)	Yes
USA 1991 [135]	Single centre sternal wound infections	8/8	RFLP (computer aided)	Outbreak isolates (n = 6 from patients, n = 2 from scrub nurse) had identical bands (≥95%); control isolates (n = 9) demonstrated band similarity of 13–53%	Yes, link between a scrub nurse and infected patients

BMT, bone marrow transplant; DNA, deoxyribonucleic acid; HCW, healthcare worker; HD, haemodialysis; ICU, intensive care unit; MLST, multilocus sequence typing; NICU, neonatal intensive care unit; PCR, polymerase chain reaction; PFGE, pulsed field gel electrophoresis; RAPD, random amplified polymorphic DNA analysis; RFLP, restriction fragment length polymorphism; TPN, total parenteral nutrition; VIP, very important person; WGS, whole genome sequencing; * These are reports of different analyses of isolates from the same outbreak.

**Table 4:** Antifungal drug therapy in patients with invasive fungal disease due to *Candida* spp., *Cryptococcus* spp., uncommon yeasts, *Aspergillus* spp., uncommon moulds, *Pneumocystis jirovecii*, and endemic fungi according to causative pathogen (adapted from published guidelines [4,194,263,332,333,334,335]).

Pathogen	First Line (Preferred) Antifungal Agent	Alternate Antifungal Agent	Antifungal Drugs to Avoid
*Candida* spp. *			
Candidemia (prior to susceptibility testing results) [335,336,337,338,339,340]	Echinocandin	FLU ^#^ (or other broad-spectrum azole); refer to in vitro susceptibility results	FLU as initial therapy in critically ill and neutropenic patients
Other forms of invasive candidiasis +/− candidemia, e.g., endocarditis, intraabdominal candidiasis [335,336,337,338,339,340]	Various: preferred agent(s) will vary with site of infection (seek Infectious Diseases advice); combination therapy may be appropriate	Various; will vary with site of infection; seek Infectious Diseases advice	-
*Cryptococcus* spp.			
CNS disease and disseminated infection (all patients) [332]	L-AmB + 5FC initial therapy followed by FLU ^#^	AmB-d + 5-FC or L-AmB only or 5-FC + FLU followed by FLU	FLU monotherapy; Echinocandins
Pulmonary infection only: Severe disease and/or large cryptococcomas (>2 cm diameter) [332]	As for CNS cryptococcosis		Echinocandins
Pulmonary infection only: Mild or asymptomatic pulmonary cryptococcosis (e.g., solitary nodules, <2 cm diameter) and without crypotcoccomas [332]	FLU	Alternative broad-spectrum azole	Echinocandins
Uncommon yeasts			
*Saprochaete*/*Magnusiomyces* spp. [4]	L-AmB +/− 5FC	VRC	Echinocandins
R*hodotorula* spp. [4]	L-AmB +/− 5-FC	AmB-d +/− 5-FC	Triazoles, echinocandins
*Trichosporon* spp. [4]	VRC or POS	FLU or POS	Echinocandins
Other			
*Pneumocystis jirovecii* [341]	TMP-SMX ^$^	Clindamycin plus primaquine; or Dapsone plus TMP	Although intravenous pentamidine has had efficacy against PCP in HIV-infected persons, survival rates were significantly lower compared with TMP-SMX and clindamycin-primaquine [342]
Moulds			
*Aspergillus* spp. [333]	VRC	POS or ISA	AmB-d **
*Lomentospora* spp. [263,334]	VRC + TRB	VRC	L-AmB, AmB-d
*Scedosporium* spp. [263,334]	VRC	VRC + L-AmB/ echinocandin/TRB	L-AmB, AmB-d
*Fusarium* spp. Complex [263,334]	VRC +/− L-AmB	L-AmB	Amb-d
Mucorales [263]	L-AmB	POS or ISA	AmB-d
Dematiaceous fungi, e.g., *Exserohilum* spp. [263]	VRC +/− L-AmB	L-AmB + triazole other than VRC	Amb-d
Endemic mycoses			
*Blastomyces* spp. [194]	L-AmB followed by ITR		
*Coccidioiddes* spp. [194]	L-AmB followed by azole		
*Histoplasma* spp. [194]	L-AmB followed by ITR		
*Sporothrix* spp. [194]	L-AmB +/− ITR		

Abbreviations: 5-FC, 5 flucytosine; AmB-d, amphotericin deoxycholate; CNS, central nervous system; FLU, fluconazole; HIV, human immunodeficiency virus; ISA, isavuconazole; ITR, itraconazole; L-AmB, liposomal amphotericin B; PCP, pneumocystis jirovecii pneumonia; POS, posaconazole; TMP, trimethoprim; TMP-SMX, trimethroprim-sulphamethoxazole; TRB, terbinafine; VRC, voriconazole; *Antifungal susceptibility testing should always be performed in invasive *Candida* infections; ** Except may be used in neonates; ^#^ alternate triazoles may be used after expert consultation; ^$^ Consider desensitization in all patients with allergy to TMP-SMX as clinically indicated and after expert consultation [341].

## Data Availability

No new data were created or analyzed in this study. Data sharing is not applicable to this article.

## References

[1] Caceres D.H., Mohd Tap R., Alastruey-Izquierdo A., Hagen F. (2020). Detection and Control of Fungal Outbreaks. Mycopathologia.

[2] Kidd S.E., Chen S.C., Meyer W., Halliday C.L. (2019). A New Age in Molecular Diagnostics for Invasive Fungal Disease: Are We Ready?. Front. Microbiol..

[3] Kidd S.E., Crawford L.C., Halliday C.L. (2021). Antifungal Susceptibility Testing and Identification. Infect. Dis. Clin. N. Am..

[4] Chen S.C., Perfect J., Colombo A.L., Cornely O.A., Groll A.H., Seidel D., Albus K., de Almedia J.N., Garcia-Effron G., Gilroy N. (2021). Global guideline for the diagnosis and management of rare yeast infections: An initiative of the ECMM in cooperation with ISHAM and ASM. Lancet Infect. Dis..

[5] Guarner J., Brandt M.E. (2011). Histopathologic diagnosis of fungal infections in the 21st century. Clin. Microbiol. Rev..

[6] White P.L., Alanio A., Brown L., Cruciani M., Hagen F., Gorton R., Lackner M., Millon L., Morton C.O., Rautemaa-Richardson R. (2022). An overview of using fungal DNA for the diagnosis of invasive mycoses. Expert Rev. Mol. Diagn..

[7] Sabino R., Wiederhold N. (2022). Diagnosis from Tissue: Histology and Identification. J. Fungi.

[8] Sangoi A.R., Rogers W.M., Longacre T.A., Montoya J.G., Baron E.J., Banaei N. (2009). Challenges and pitfalls of morphologic identification of fungal infections in histologic and cytologic specimens: A ten-year retrospective review at a single institution. Am. J. Clin. Pathol..

[9] Shah A.A., Hazen K.C. (2013). Diagnostic accuracy of histopathologic and cytopathologic examination of *Aspergillus* species. Am. J. Clin. Pathol..

[10] Kung V.L., Chernock R.D., Burnham C.D. (2018). Diagnostic accuracy of fungal identification in histopathology and cytopathology specimens. Eur. J. Clin. Microbiol. Infect. Dis..

[11] Lockhart S.R., Bialek R., Kibbler C.C., Cuenca-Estrella M., Jensen H.E., Kontoyiannis D.P. (2021). Molecular Techniques for Genus and Species Determination of Fungi From Fresh and Paraffin-Embedded Formalin-Fixed Tissue in the Revised EORTC/MSGERC Definitions of Invasive Fungal Infection. Clin. Infect. Dis..

[12] Lagrou K., Chen S., Masur H., Viscoli C., Decker C.F., Pagano L., Groll A.H. (2021). *Pneumocystis jirovecii* Disease: Basis for the Revised EORTC/MSGERC Invasive Fungal Disease Definitions in Individuals without Human Immunodeficiency Virus. Clin. Infect. Dis..

[13] Apostolopoulou A., Fishman J.A. (2022). The Pathogenesis and Diagnosis of *Pneumocystis jiroveci* Pneumonia. J. Fungi.

[14] Alanio A., Hauser P.M., Lagrou K., Melchers W.J., Helweg-Larsen J., Matos O., Cesaro S., Maschmeyer G., Einsele H., Donnelly J.P. (2016). ECIL guidelines for the diagnosis of *Pneumocystis jirovecii* pneumonia in patients with haematological malignancies and stem cell transplant recipients. J. Antimicrob. Chemother..

[15] Del Corpo O., Butler-Laporte G., Sheppard D.C., Cheng M.P., McDonald E.G., Lee T.C. (2020). Diagnostic accuracy of serum (1-3)-beta-D-glucan for *Pneumocystis jirovecii* pneumonia: A systematic review and meta-analysis. Clin. Microbiol. Infect..

[16] McCarty T.P., White C.M., Pappas P.G. (2021). Candidemia and Invasive Candidiasis. Infect. Dis. Clin. N. Am..

[17] Lass-Florl C. (2009). Zygomycosis: Conventional laboratory diagnosis. Clin. Microbiol. Infect..

[18] Borman A.M., Fraser M., Johnson E.M. (2021). CHROMagar^TM^ Candida Plus: A novel chromogenic agar that permits the rapid identification of *Candida auris*. Med. Mycol..

[19] Mulet Bayona J.V., Salvador Garcia C., Tormo Palop N., Valentin Martin A., Gonzalez Padron C., Colomina Rodriguez J., Peman J., Gimeno Cardona C. (2022). Novel Chromogenic Medium CHROMagar(TM) Candida Plus for Detection of *Candida auris* and Other Candida Species from Surveillance and Environmental Samples: A Multicenter Study. J. Fungi.

[20] Welsh R.M., Bentz M.L., Shams A., Houston H., Lyons A., Rose L.J., Litvintseva A.P. (2017). Survival, Persistence, and Isolation of the Emerging Multidrug-Resistant Pathogenic Yeast *Candida auris* on a Plastic Health Care Surface. J. Clin. Microbiol..

[21] Zeller I., Schabereiter-Gurtner C., Mihalits V., Selitsch B., Barousch W., Hirschl A.M., Makristathis A., Willinger B. (2017). Detection of fungal pathogens by a new broad range real-time PCR assay targeting the fungal ITS2 region. J. Med. Microbiol..

[22] Gade L., Scheel C.M., Pham C.D., Lindsley M.D., Iqbal N., Cleveland A.A., Whitney A.M., Lockhart S.R., Brandt M.E., Litvintseva A.P. (2013). Detection of fungal DNA in human body fluids and tissues during a multistate outbreak of fungal meningitis and other infections. Eukaryot. Cell.

[23] Zhao Y., Armeanu E., DiVerniero R., Lewis T.A., Dobson R.C., Kontoyiannis D.P., Roilides E., Walsh T.J., Perlin D.S. (2014). Fungal DNA detected in blood samples of patients who received contaminated methylprednisolone injections reveals increased complexity of causative agents. J. Clin. Microbiol..

[24] Cruciani M., Mengoli C., Barnes R., Donnelly J.P., Loeffler J., Jones B.L., Klingspor L., Maertens J., Morton C.O., White L.P. (2019). Polymerase chain reaction blood tests for the diagnosis of invasive aspergillosis in immunocompromised people. Cochrane Database Syst. Rev..

[25] White P.L., Bretagne S., Caliendo A.M., Loeffler J., Patterson T.F., Slavin M., Wingard J.R. (2021). *Aspergillus* Polymerase Chain Reaction-An Update on Technical Recommendations, Clinical Applications, and Justification for Inclusion in the Second Revision of the EORTC/MSGERC Definitions of Invasive Fungal Disease. Clin. Infect. Dis..

[26] Springer J., Lackner M., Ensinger C., Risslegger B., Morton C.O., Nachbaur D., Lass-Florl C., Einsele H., Heinz W.J., Loeffler J. (2016). Clinical evaluation of a Mucorales-specific real-time PCR assay in tissue and serum samples. J. Med. Microbiol..

[27] Springer J., White P.L., Kessel J., Wieters I., Teschner D., Korczynski D., Liebregts T., Cornely O.A., Schwartz S., Elgeti T. (2018). A Comparison of *Aspergillus* and Mucorales PCR Testing of Different Bronchoalveolar Lavage Fluid Fractions from Patients with Suspected Invasive Pulmonary Fungal Disease. J. Clin. Microbiol..

[28] Millon L., Caillot D., Berceanu A., Bretagne S., Lanternier F., Morio F., Letscher-Bru V., Dalle F., Denis B., Alanio A. (2022). Evaluation of Serum Mucorales Polymerase Chain Reaction (PCR) for the Diagnosis of Mucormycoses: The MODIMUCOR Prospective Trial. Clin. Infect. Dis..

[29] Gade L., Grgurich D.E., Kerkering T.M., Brandt M.E., Litvintseva A.P. (2015). Utility of real-time PCR for detection of Exserohilum rostratum in body and tissue fluids during the multistate outbreak of fungal meningitis and other infections. J. Clin. Microbiol..

[30] Phipps L.M., Chen S.C., Kable K., Halliday C.L., Firacative C., Meyer W., Wong G., Nankivell B.J. (2011). Nosocomial *Pneumocystis jirovecii* pneumonia: Lessons from a cluster in kidney transplant recipients. Transplantation.

[31] Robin C., Alanio A., Gits-Muselli M., la Martire G., Schlemmer F., Botterel F., Angebault C., Leclerc M., Beckerich F., Redjoul R. (2017). Molecular Demonstration of a Pneumocystis Outbreak in Stem Cell Transplant Patients: Evidence for Transmission in the Daycare Center. Front. Microbiol..

[32] Vindrios W., Argy N., Le Gal S., Lescure F.X., Massias L., Le M.P., Wolff M., Yazdanpanah Y., Nevez G., Houze S. (2017). Outbreak of *Pneumocystis jirovecii* Infection Among Heart Transplant Recipients: Molecular Investigation and Management of an Interhuman Transmission. Clin. Infect. Dis..

[33] Avni T., Leibovici L., Paul M. (2011). PCR diagnosis of invasive candidiasis: Systematic review and meta-analysis. J. Clin. Microbiol..

[34] Donnelly J.P., Chen S.C., Kauffman C.A., Steinbach W.J., Baddley J.W., Verweij P.E., Clancy C.J., Wingard J.R., Lockhart S.R., Groll A.H. (2020). Revision and Update of the Consensus Definitions of Invasive Fungal Disease from the European Organization for Research and Treatment of Cancer and the Mycoses Study Group Education and Research Consortium. Clin. Infect. Dis..

[35] Thompson G.R., Boulware D.R., Bahr N.C., Clancy C.J., Harrison T.S., Kauffman C.A., Le T., Miceli M.H., Mylonakis E., Nguyen M.H. (2022). Noninvasive Testing and Surrogate Markers in Invasive Fungal Diseases. Open Forum Infect. Dis..

[36] Lockhart S.R., Lyman M.M., Sexton D.J. (2022). Tools for Detecting a “Superbug”: Updates on *Candida auris* Testing. J. Clin. Microbiol..

[37] Leeflang M.M., Debets-Ossenkopp Y.J., Wang J., Visser C.E., Scholten R.J., Hooft L., Bijlmer H.A., Reitsma J.B., Zhang M., Bossuyt P.M. (2015). Galactomannan detection for invasive aspergillosis in immunocompromised patients. Cochrane Database Syst. Rev..

[38] Mercier T., Castagnola E., Marr K.A., Wheat L.J., Verweij P.E., Maertens J.A. (2021). Defining Galactomannan Positivity in the Updated EORTC/MSGERC Consensus Definitions of Invasive Fungal Diseases. Clin. Infect. Dis..

[39] de Heer K., Gerritsen M.G., Visser C.E., Leeflang M.M. (2019). Galactomannan detection in broncho-alveolar lavage fluid for invasive aspergillosis in immunocompromised patients. Cochrane Database Syst. Rev..

[40] Zou M., Tang L., Zhao S., Zhao Z., Chen L., Chen P., Huang Z., Li J., Chen L., Fan X. (2012). Systematic review and meta-analysis of detecting galactomannan in bronchoalveolar lavage fluid for diagnosing invasive aspergillosis. PLoS ONE.

[41] Cadena J., Thompson G.R., Patterson T.F. (2021). Aspergillosis: Epidemiology, Diagnosis, and Treatment. Infect. Dis. Clin. N. Am..

[42] Thornton C.R. (2008). Development of an immunochromatographic lateral-flow device for rapid serodiagnosis of invasive aspergillosis. Clin. Vaccine Immunol..

[43] Thornton C.R. (2010). Detection of invasive aspergillosis. Adv. Appl. Microbiol..

[44] Jenks J.D., Miceli M.H., Prattes J., Mercier T., Hoenigl M. (2020). The *Aspergillus* Lateral Flow Assay for the Diagnosis of Invasive Aspergillosis: An Update. Curr. Fungal Infect. Rep..

[45] Mercier T., Dunbar A., de Kort E., Schauwvlieghe A., Reynders M., Guldentops E., Blijlevens N.M.A., Vonk A.G., Rijnders B., Verweij P.E. (2020). Lateral flow assays for diagnosing invasive pulmonary aspergillosis in adult hematology patients: A comparative multicenter study. Med. Mycol..

[46] Jenks J.D., Mehta S.R., Taplitz R., Law N., Reed S.L., Hoenigl M. (2019). Bronchoalveolar lavage *Aspergillus* Galactomannan lateral flow assay versus *Aspergillus*-specific lateral flow device test for diagnosis of invasive pulmonary Aspergillosis in patients with hematological malignancies. J. Infect..

[47] Lass-Florl C., Lo Cascio G., Nucci M., Camargo Dos Santos M., Colombo A.L., Vossen M., Willinger B. (2019). Respiratory specimens and the diagnostic accuracy of *Aspergillus* lateral flow assays (LFA-IMMY): Real-life data from a multicentre study. Clin. Microbiol. Infect..

[48] Lamoth F., Akan H., Andes D., Cruciani M., Marchetti O., Ostrosky-Zeichner L., Racil Z., Clancy C.J. (2021). Assessment of the Role of 1,3-beta-d-Glucan Testing for the Diagnosis of Invasive Fungal Infections in Adults. Clin. Infect. Dis..

[49] Friedrich R., Rappold E., Bogdan C., Held J. (2018). Comparative Analysis of the Wako beta-Glucan Test and the Fungitell Assay for Diagnosis of Candidemia and *Pneumocystis jirovecii* Pneumonia. J. Clin. Microbiol..

[50] Onishi A., Sugiyama D., Kogata Y., Saegusa J., Sugimoto T., Kawano S., Morinobu A., Nishimura K., Kumagai S. (2012). Diagnostic accuracy of serum 1,3-beta-D-glucan for *Pneumocystis jiroveci* pneumonia, invasive candidiasis, and invasive aspergillosis: Systematic review and meta-analysis. J. Clin. Microbiol..

[51] Sun R., Lv D., Xiao M., Zhang L., Xu J., Yu X., Zhu H., Yang J. (2021). Diagnostic accuracy of the 1,3-beta-D-glucan test and lactate dehydrogenase for pneumocystis pneumonia in non-HIV patients. Sci. Rep..

[52] Finkelman M.A. (2020). Specificity Influences in (1-->3)-beta-d-Glucan-Supported Diagnosis of Invasive Fungal Disease. J. Fungi.

[53] Litvintseva A.P., Lindsley M.D., Gade L., Smith R., Chiller T., Lyons J.L., Thakur K.T., Zhang S.X., Grgurich D.E., Kerkering T.M. (2014). Utility of (1-3)-beta-D-glucan testing for diagnostics and monitoring response to treatment during the multistate outbreak of fungal meningitis and other infections. Clin. Infect. Dis..

[54] Dupont D., Normand A.C., Persat F., Hendrickx M., Piarroux R., Wallon M. (2019). Comparison of matrix-assisted laser desorption ionization time of flight mass spectrometry (MALDI-TOF MS) systems for the identification of moulds in the routine microbiology laboratory. Clin. Microbiol. Infect..

[55] Fraser M., Brown Z., Houldsworth M., Borman A.M., Johnson E.M. (2016). Rapid identification of 6328 isolates of pathogenic yeasts using MALDI-ToF MS and a simplified, rapid extraction procedure that is compatible with the Bruker Biotyper platform and database. Med. Mycol..

[56] Lau A.F., Drake S.K., Calhoun L.B., Henderson C.M., Zelazny A.M. (2013). Development of a clinically comprehensive database and a simple procedure for identification of molds from solid media by matrix-assisted laser desorption ionization-time of flight mass spectrometry. J. Clin. Microbiol..

[57] Patel R. (2019). A Moldy Application of MALDI: MALDI-ToF Mass Spectrometry for Fungal Identification. J. Fungi.

[58] Wang H., Fan Y.Y., Kudinha T., Xu Z.P., Xiao M., Zhang L., Fan X., Kong F., Xu Y.C. (2016). A Comprehensive Evaluation of the Bruker Biotyper MS and Vitek MS Matrix-Assisted Laser Desorption Ionization-Time of Flight Mass Spectrometry Systems for Identification of Yeasts, Part of the National China Hospital Invasive Fungal Surveillance Net (CHIF-NET) Study, 2012 to 2013. J. Clin. Microbiol..

[59] Wilkendorf L.S., Bowles E., Buil J.B., van der Lee H.A.L., Posteraro B., Sanguinetti M., Verweij P.E. (2020). Update on Matrix-Assisted Laser Desorption Ionization-Time of Flight Mass Spectrometry Identification of Filamentous Fungi. J. Clin. Microbiol..

[60] Irinyi L., Serena C., Garcia-Hermoso D., Arabatzis M., Desnos-Ollivier M., Vu D., Cardinali G., Arthur I., Normand A.C., Giraldo A. (2015). International Society of Human and Animal Mycology (ISHAM)-ITS reference DNA barcoding database—The quality controlled standard tool for routine identification of human and animal pathogenic fungi. Med. Mycol..

[61] Meyer W., Irinyi L., Hoang M.T.V., Robert V., Garcia-Hermoso D., Desnos-Ollivier M., Yurayart C., Tsang C.C., Lee C.Y., Woo P.C.Y. (2019). Database establishment for the secondary fungal DNA barcode translational elongation factor 1alpha (TEF1alpha) (1). Genome.

[62] Schoch C.L., Seifert K.A., Huhndorf S., Robert V., Spouge J.L., Levesque C.A., Chen W., Fungal Barcoding C., Bolchacova E., Fungal Barcoding Consortium Author List (2012). Nuclear ribosomal internal transcribed spacer (ITS) region as a universal DNA barcode marker for Fungi. Proc. Natl. Acad. Sci. USA.

[63] Balajee S.A., Borman A.M., Brandt M.E., Cano J., Cuenca-Estrella M., Dannaoui E., Guarro J., Haase G., Kibbler C.C., Meyer W. (2009). Sequence-based identification of *Aspergillus*, *Fusarium*, and mucorales species in the clinical mycology laboratory: Where are we and where should we go from here?. J. Clin. Microbiol..

[64] Hoang M.T.V., Irinyi L., Chen S.C.A., Sorrell T.C., Meyer W., the ISHAM Barcoding of Medical Fungi Working Group (2019). Dual DNA Barcoding for the Molecular Identification of the Agents of Invasive Fungal Infections. Front. Microbiol..

[65] Clinical and Laboratory Standards Institute (2018). Interpretive Criteria for Identification of Bacteria and Fungi by Targeted DNA Sequencing.

[66] Ioakimidou A., Vyzantiadis T.A., Sakellari I., Arabatzis M., Smias C., Douka V., Velegraki A., Anagnostopoulos A., Malissiovas N. (2013). An unusual cluster of Acremonium kiliense fungaemias in a haematopoietic cell transplantation unit. Diagn. Microbiol. Infect. Dis..

[67] Singh A., Goering R.V., Simjee S., Foley S.L., Zervos M.J. (2006). Application of molecular techniques to the study of hospital infection. Clin. Microbiol. Rev..

[68] Gil-Lamaignere C., Roilides E., Hacker J., Muller F.M. (2003). Molecular typing for fungi—A critical review of the possibilities and limitations of currently and future methods. Clin. Microbiol. Infect..

[69] Bernhardt A., Sedlacek L., Wagner S., Schwarz C., Wurstl B., Tintelnot K. (2013). Multilocus sequence typing of *Scedosporium apiospermum* and *Pseudallescheria boydii* isolates from cystic fibrosis patients. J. Cyst. Fibros..

[70] Harun A., Kan A., Schwabenbauer K., Gilgado F., Perdomo H., Firacative C., Losert H., Abdullah S., Giraud S., Kaltseis J. (2021). Multilocus Sequence Typing Reveals Extensive Genetic Diversity of the Emerging Fungal Pathogen *Scedosporium* aurantiacum. Front. Cell. Infect. Microbiol..

[71] Pasic L., Goterris L., Guerrero-Murillo M., Irinyi L., Kan A., Ponce C.A., Vargas S.L., Martin-Gomez M.T., Meyer W. (2020). Consensus Multilocus Sequence Typing Scheme for *Pneumocystis jirovecii*. J. Fungi.

[72] Ashu E.E., Hagen F., Chowdhary A., Meis J.F., Xu J. (2017). Global Population Genetic Analysis of *Aspergillus* fumigatus. mSphere.

[73] Vatanshenassan M., Boekhout T., Mauder N., Robert V., Maier T., Meis J.F., Berman J., Then E., Kostrzewa M., Hagen F. (2020). Evaluation of Microsatellite Typing, ITS Sequencing, AFLP Fingerprinting, MALDI-TOF MS, and Fourier-Transform Infrared Spectroscopy Analysis of *Candida auris*. J. Fungi.

[74] Wu Y., Zhou H.J., Che J., Li W.G., Bian F.N., Yu S.B., Zhang L.J., Lu J. (2014). Multilocus microsatellite markers for molecular typing of *Candida tropicalis* isolates. BMC Microbiol..

[75] Gits-Muselli M., Peraldi M.N., de Castro N., Delcey V., Menotti J., Guigue N., Hamane S., Raffoux E., Bergeron A., Valade S. (2015). New Short Tandem Repeat-Based Molecular Typing Method for *Pneumocystis jirovecii* Reveals Intrahospital Transmission between Patients from Different Wards. PLoS ONE.

[76] Vallabhaneni S., Kallen A., Tsay S., Chow N., Welsh R., Kerins J., Kemble S.K., Pacilli M., Black S.R., Landon E. (2017). Investigation of the First Seven Reported Cases of *Candida auris*, a Globally Emerging Invasive, Multidrug-Resistant Fungus-United States, May 2013–August 2016. Am. J. Transplant..

[77] Eyre D.W., Sheppard A.E., Madder H., Moir I., Moroney R., Quan T.P., Griffiths D., George S., Butcher L., Morgan M. (2018). A *Candida auris* Outbreak and Its Control in an Intensive Care Setting. N. Engl. J. Med..

[78] Almaghrabi R.S., Albalawi R., Mutabagani M., Atienza E., Aljumaah S., Gade L., Forsberg K., Litvintseva A., Althawadi S. (2020). Molecular characterisation and clinical outcomes of *Candida auris* infection: Single-centre experience in Saudi Arabia. Mycoses.

[79] Theodoropoulos N.M., Bolstorff B., Bozorgzadeh A., Brandeburg C., Cumming M., Daly J.S., Ellison R.T., Forsberg K., Gade L., Gibson L. (2020). *Candida auris* outbreak involving liver transplant recipients in a surgical intensive care unit. Am. J. Transplant..

[80] Di Pilato V., Codda G., Ball L., Giacobbe D.R., Willison E., Mikulska M., Magnasco L., Crea F., Vena A., Pelosi P. (2021). Molecular Epidemiological Investigation of a Nosocomial Cluster of *C. auris*: Evidence of Recent Emergence in Italy and Ease of Transmission during the COVID-19 Pandemic. J. Fungi.

[81] Tse H., Tsang A.K.L., Chu Y.W., Tsang D.N.C. (2021). Draft Genome Sequences of 19 Clinical Isolates of *Candida auris* from Hong Kong. Microbiol. Resour. Announc..

[82] Hanson B.M., Dinh A.Q., Tran T.T., Arenas S., Pronty D., Gershengorn H.B., Ferreira T., Arias C.A., Shukla B.S. (2021). *Candida auris* Invasive Infections during a COVID-19 Case Surge. Antimicrob. Agents Chemother..

[83] Roberts S.C., Zembower T.R., Ozer E.A., Qi C. (2021). Genetic Evaluation of Nosocomial *Candida auris* Transmission. J. Clin. Microbiol..

[84] Karmarkar E.N., O’Donnell K., Prestel C., Forsberg K., Gade L., Jain S., Schan D., Chow N., McDermott D., Rossow J. (2021). Rapid Assessment and Containment of *Candida auris* Transmission in Postacute Care Settings-Orange County, California, 2019. Ann. Intern. Med..

[85] Eckbo E.J., Wong T., Bharat A., Cameron-Lane M., Hoang L., Dawar M., Charles M. (2021). First reported outbreak of the emerging pathogen *Candida auris* in Canada. Am. J. Infect. Control.

[86] Taori S.K., Rhodes J., Khonyongwa K., Szendroi A., Smith M., Borman A.M., Kumarage J., Brown C.S., Moore G., Desai N. (2022). First experience of implementing *Candida auris* real-time PCR for surveillance in the UK: Detection of multiple introductions with two international clades and improved patient outcomes. J. Hosp. Infect..

[87] Alanio A., Snell H.M., Cordier C., Desnos-Olivier M., Dellière S., Aissaoui N., Sturny-Leclère A., Da Silva E., Eblé C., Rouveau M. (2022). First Patient-to-Patient Intrahospital Transmission of Clade I *Candida auris* in France Revealed after a Two-Month Incubation Period. Microbiol. Spectr..

[88] Spruijtenburg B., Badali H., Abastabar M., Mirhendi H., Khodavaisy S., Sharifisooraki J., Taghizadeh Armaki M., de Groot T., Meis J.F. (2022). Confirmation of fifth *Candida auris* clade by whole genome sequencing. Emerg. Microbes Infect..

[89] Codda G., Willison E., Magnasco L., Morici P., Giacobbe D.R., Mencacci A., Marini D., Mikulska M., Bassetti M., Marchese A. (2023). In vivo evolution to echinocandin resistance and increasing clonal heterogeneity in *Candida auris* during a difficult-to-control hospital outbreak, Italy, 2019 to 2022. Eurosurveillance.

[90] Ben Abid F., Salah H., Sundararaju S., Dalil L., Abdelwahab A.H., Salameh S., Ibrahim E.B., Almaslmani M.A., Tang P., Perez-Lopez A. (2023). Molecular characterization of *Candida auris* outbreak isolates in Qatar from patients with COVID-19 reveals the emergence of isolates resistant to three classes of antifungal drugs. Clin. Microbiol. Infect..

[91] Calvo B., Melo A.S., Perozo-Mena A., Hernandez M., Francisco E.C., Hagen F., Meis J.F., Colombo A.L. (2016). First report of *Candida auris* in America: Clinical and microbiological aspects of 18 episodes of candidemia. J. Infect..

[92] Schelenz S., Hagen F., Rhodes J.L., Abdolrasouli A., Chowdhary A., Hall A., Ryan L., Shackleton J., Trimlett R., Meis J.F. (2016). First hospital outbreak of the globally emerging *Candida auris* in a European hospital. Antimicrob. Resist. Infect. Control.

[93] Ruiz-Gaitan A., Moret A.M., Tasias-Pitarch M., Aleixandre-Lopez A.I., Martinez-Morel H., Calabuig E., Salavert-Lleti M., Ramirez P., Lopez-Hontangas J.L., Hagen F. (2018). An outbreak due to *Candida auris* with prolonged colonisation and candidaemia in a tertiary care European hospital. Mycoses.

[94] Alfouzan W., Ahmad S., Dhar R., Asadzadeh M., Almerdasi N., Abdo N.M., Joseph L., de Groot T., Alali W.Q., Khan Z. (2020). Molecular Epidemiology of *Candida auris* Outbreak in a Major Secondary-Care Hospital in Kuwait. J. Fungi.

[95] Nobrega de Almeida J., Brandão I.B., Francisco E.C., de Almeida S.L.R., de Oliveira Dias P., Pereira F.M., Santos Ferreira F., de Andrade T.S., de Miranda Costa M.M., de Souza Jordão R.T. (2021). Axillary Digital Thermometers uplifted a multidrug-susceptible *Candida auris* outbreak among COVID-19 patients in Brazil. Mycoses.

[96] Araujo R., Sampaio-Maia B. (2018). Fungal Genomes and Genotyping. Adv. Appl. Microbiol..

[97] Ma L.J., Ibrahim A.S., Skory C., Grabherr M.G., Burger G., Butler M., Elias M., Idnurm A., Lang B.F., Sone T. (2009). Genomic analysis of the basal lineage fungus *Rhizopus oryzae* reveals a whole-genome duplication. PLoS Genet..

[98] Salem-Bango Z., Price T.K., Chan J.L., Chandrasekaran S., Garner O.B., Yang S. (2023). Fungal Whole-Genome Sequencing for Species Identification: From Test Development to Clinical Utilization. J. Fungi.

[99] Desnos-Ollivier M., Fekkar A., Bretagne S. (2021). Earliest case of *Candida auris* infection imported in 2007 in Europe from India prior to the 2009 description in Japan. J. Med. Mycol..

[100] Bougnoux M.E., Brun S., Zahar J.R. (2018). Healthcare-associated fungal outbreaks: New and uncommon species, New molecular tools for investigation and prevention. Antimicrob. Resist. Infect. Control.

[101] Litvintseva A.P., Brandt M.E., Mody R.K., Lockhart S.R. (2015). Investigating fungal outbreaks in the 21st century. PLoS Pathog..

[102] Tsang C.C., Teng J.L.L., Lau S.K.P., Woo P.C.Y. (2021). Rapid Genomic Diagnosis of Fungal Infections in the Age of Next-Generation Sequencing. J. Fungi.

[103] Guinea J., Mezquita S., Gomez A., Padilla B., Zamora E., Sanchez-Luna M., Sanchez-Carrillo C., Munoz P., Escribano P. (2021). Whole genome sequencing confirms *Candida albicans* and *Candida parapsilosis* microsatellite sporadic and persistent clones causing outbreaks of candidemia in neonates. Med. Mycol..

[104] Brillowska-Dabrowska A., Schon T., Pannanusorn S., Lonnbro N., Bernhoff L., Bonnedal J., Haggstrom J., Wistedt A., Fernandez V., Arendrup M.C. (2009). A nosocomial outbreak of *Candida parapsilosis* in southern Sweden verified by genotyping. Scand. J. Infect. Dis..

[105] Vaz C., Sampaio P., Clemons K.V., Huang Y.C., Stevens D.A., Pais C. (2011). Microsatellite multilocus genotyping clarifies the relationship of *Candida parapsilosis* strains involved in a neonatal intensive care unit outbreak. Diagn. Microbiol. Infect. Dis..

[106] Diab-Elschahawi M., Forstner C., Hagen F., Meis J.F., Lassnig A.M., Presterl E., Klaassen C.H. (2012). Microsatellite genotyping clarified conspicuous accumulation of *Candida parapsilosis* at a cardiothoracic surgery intensive care unit. J. Clin. Microbiol..

[107] da Silva Ruiz L., Montelli A.C., Sugizaki Mde F., Da Silva E.G., De Batista G.C., Moreira D., Paula C.R. (2013). Outbreak of fungemia caused by *Candida parapsilosis* in a neonatal intensive care unit: Molecular investigation through microsatellite analysis. Rev. Iberoam. Micol..

[108] Wang H., Zhang L., Kudinha T., Kong F., Ma X.J., Chu Y.Z., Kang M., Sun Z.Y., Li R.Y., Liao K. (2016). Investigation of an unrecognized large-scale outbreak of *Candida parapsilosis* sensu stricto fungaemia in a tertiary-care hospital in China. Sci. Rep..

[109] Cala C., Fontana I., Di Carlo P., Mascarella C., Fasciana T., Reale S., Sergi C., Giammanco A. (2020). *Candida parapsilosis* Infection: A Multilocus Microsatellite Genotyping-Based Survey Demonstrating an Outbreak in Hospitalized Patients. Ann. Clin. Lab. Sci..

[110] Arastehfar A., Hilmioğlu-Polat S., Daneshnia F., Pan W., Hafez A., Fang W., Liao W., Şahbudak-Bal Z., Metin D.Y., Júnior J.N.D.A. (2021). Clonal Candidemia Outbreak by *Candida parapsilosis* Carrying Y132F in Turkey: Evolution of a Persisting Challenge. Front. Cell. Infect. Microbiol..

[111] Corzo-Leon D.E., Peacock M., Rodriguez-Zulueta P., Salazar-Tamayo G.J., MacCallum D.M. (2021). General hospital outbreak of invasive candidiasis due to azole-resistant *Candida parapsilosis* associated with an Erg11 Y132F mutation. Med. Mycol..

[112] Fekkar A., Blaize M., Bouglé A., Normand A.C., Raoelina A., Kornblum D., Kamus L., Piarroux R., Imbert S. (2021). Hospital outbreak of fluconazole-resistant *Candida parapsilosis*: Arguments for clonal transmission and long-term persistence. Antimicrob. Agents Chemother..

[113] Miyake A., Gotoh K., Iwahashi J., Togo A., Horita R., Miura M., Kinoshita M., Ohta K., Yamashita Y., Watanabe H. (2022). Characteristics of Biofilms Formed by *C. parapsilosis* Causing an Outbreak in a Neonatal Intensive Care Unit. J. Fungi.

[114] Thomaz D.Y., Del Negro G.M.B., Ribeiro L.B., da Silva M., Carvalho G., Camargo C.H., de Almeida J.N., Motta A.L., Siciliano R.F., Sejas O.N.E. (2022). A Brazilian Inter-Hospital Candidemia Outbreak Caused by Fluconazole-Resistant *Candida parapsilosis* in the COVID-19 Era. J. Fungi.

[115] Daneshnia F., de Almeida Junior J.N., Arastehfar A., Lombardi L., Shor E., Moreno L., Verena Mendes A., Goreth Barberino M., Thomaz Yamamoto D., Butler G. (2022). Determinants of fluconazole resistance and echinocandin tolerance in *C. parapsilosis* isolates causing a large clonal candidemia outbreak among COVID-19 patients in a Brazilian ICU. Emerg. Microbes Infect..

[116] Hare R.K., Arastehfar A., Rosendahl S., Charsizadeh A., Daneshnia F., Eshaghi H., Mirhendi H., Boekhout T., Hagen F., Arendrup M.C. (2022). Candidemia among Hospitalized Pediatric Patients Caused by Several Clonal Lineages of *Candida parapsilosis*. J. Fungi.

[117] Mohammad N., Normand A.C., Nabet C., Godmer A., Brossas J.Y., Blaize M., Bonnal C., Fekkar A., Imbert S., Tannier X. (2023). Improving the Detection of Epidemic Clones in *Candida parapsilosis* Outbreaks by Combining MALDI-TOF Mass Spectrometry and Deep Learning Approaches. Microorganisms.

[118] Presente S., Bonnal C., Normand A.C., Gaudonnet Y., Fekkar A., Timsit J.F., Kernéis S. (2023). Hospital Clonal Outbreak of Fluconazole-Resistant *Candida parapsilosis* Harboring the Y132F ERG11p Substitution in a French Intensive Care Unit. Antimicrob. Agents Chemother..

[119] Haciseyitoglu D., Cag Y. (2019). An outbreak of candidemia due to *Candida parapsilosis* in an adult intensive care unit. Infez. Med..

[120] Clark T.A., Slavinski S.A., Morgan J., Lott T., Arthington-Skaggs B.A., Brandt M.E., Webb R.M., Currier M., Flowers R.H., Fridkin S.K. (2004). Epidemiologic and molecular characterization of an outbreak of *Candida parapsilosis* bloodstream infections in a community hospital. J. Clin. Microbiol..

[121] Garcia San Miguel L., Pla J., Cobo J., Navarro F., Sanchez-Sousa A., Alvarez M.E., Martos I., Moreno S. (2004). Morphotypic and genotypic characterization of sequential *Candida parapsilosis* isolates from an outbreak in a pediatric intensive care unit. Diagn. Microbiol. Infect. Dis..

[122] van Asbeck E.C., Huang Y.C., Markham A.N., Clemons K.V., Stevens D.A. (2007). *Candida parapsilosis* fungemia in neonates: Genotyping results suggest healthcare workers hands as source, and review of published studies. Mycopathologia.

[123] Dizbay M., Kalkanci A., Sezer B.E., Aktas F., Aydogan S., Fidan I., Kustimur S., Sugita T. (2008). Molecular investigation of a fungemia outbreak due to *Candida parapsilosis* in an intensive care unit. Braz. J. Infect. Dis..

[124] Hernandez-Castro R., Arroyo-Escalante S., Carrillo-Casas E.M., Moncada-Barron D., Alvarez-Verona E., Hernandez-Delgado L., Torres-Narvaez P., Lavalle-Villalobos A. (2010). Outbreak of *Candida parapsilosis* in a neonatal intensive care unit: A health care workers source. Eur. J. Pediatr..

[125] Reissa E., Lasker B.A., Iqbal N.J., James M., Arthington-Skaggs B.A. (2008). Molecular epidemiology of *Candida parapsilosis* sepsis from outbreak investigations in neonatal intensive care units. Infect. Genet. Evol..

[126] Kuhn D.M., Mikherjee P.K., Clark T.A., Pujol C., Chandra J., Hajjeh R.A., Warnock D.W., Soil D.R., Ghannoum M.A. (2004). *Candida parapsilosis* characterization in an outbreak setting. Emerg. Infect. Dis..

[127] Welbel S.F., McNeil M.M., Kuykendall R.J., Lott T.J., Pramanik A., Silberman R., Oberle A.D., Bland L.A., Aguero S., Arduino M. (1996). *Candida parapsilosis* bloodstream infections in neonatal intensive care unit patients: Epidemiologic and laboratory confirmation of a common source outbreak. Pediatr. Infect. Dis. J..

[128] Huang Y.C., Lin T.Y., Leu H.S., Peng H.L., Wu J.H., Chang H.Y. (1999). Outbreak of *Candida parapsilosis* fungemia in neonatal intensive care units: Clinical implications and genotyping analysis. Infection.

[129] Vazquez J.A., Boikov D., Boikov S.G., Dajani A.S. (1997). Use of electrophoretic karyotyping in the evaluation of Candida infections in a neonatal intensive-care unit. Infect. Control Hosp. Epidemiol..

[130] Levin A.S., Costa S.F., Mussi N.S., Basso M., Sinto S.I., Machado C., Geiger D.C., Villares M.C., Schreiber A.Z., Barone A.A. (1998). *Candida parapsilosis* fungemia associated with implantable and semi-implantable central venous catheters and the hands of healthcare workers. Diagn. Microbiol. Infect. Dis..

[131] Jang S.J., Han H.L., Lee S.H., Ryu S.Y., Chaulagain B.P., Moon Y.L., Kim D.H., Jeong O.Y., Shin J.H., Moon D.S. (2005). PFGE-based epidemiological study of an outbreak of *Candida tropicalis* candiduria: The importance of medical waste as a reservoir of nosocomial infection. Jpn. J. Infect. Dis..

[132] Barac A., Cevik M., Colovic N., Lekovic D., Stevanovic G., Micic J., Rubino S. (2020). Investigation of a healthcare-associated *Candida tropicalis* candidiasis cluster in a haematology unit and a systematic review of nosocomial outbreaks. Mycoses.

[133] Boyce J.M., Dumigan D.G., Havill N.L., Hollis R.J., Pfaller M.A., Moore B.A. (2021). A multi-center outbreak of *Candida tropicalis* bloodstream infections associated with contaminated hemodialysis machine prime buckets. Am. J. Infect. Control.

[134] Roilides E., Farmaki E., Evdoridou J., Francesconi A., Kasai M., Filioti J., Tsivitanidou M., Sofianou D., Kremenopoulos G., Walsh T.J. (2003). *Candida tropicalis* in a neonatal intensive care unit: Epidemiologic and molecular analysis of an outbreak of infection with an uncommon neonatal pathogen. J. Clin. Microbiol..

[135] Doebbeling B.N., Hollis R.J., Isenberg H.D., Wenzel R.P., Pfaller M.A. (1991). Restriction fragment analysis of a *Candida tropicalis* outbreak of sternal wound infections. J. Clin. Microbiol..

[136] Kathuria S., Singh P.K., Sharma C., Prakash A., Masih A., Kumar A., Meis J.F., Chowdhary A. (2015). Multidrug-Resistant *Candida auris* Misidentified as Candida haemulonii: Characterization by Matrix-Assisted Laser Desorption Ionization-Time of Flight Mass Spectrometry and DNA Sequencing and Its Antifungal Susceptibility Profile Variability by Vitek 2, CLSI Broth Microdilution, and Etest Method. J. Clin. Microbiol..

[137] Rossato L., Colombo A.L. (2018). *Candida auris*: What Have We Learned About Its Mechanisms of Pathogenicity?. Front. Microbiol..

[138] Satoh K., Makimura K., Hasumi Y., Nishiyama Y., Uchida K., Yamaguchi H. (2009). *Candida auris* sp. nov., a novel ascomycetous yeast isolated from the external ear canal of an inpatient in a Japanese hospital. Microbiol. Immunol..

[139] Kim M.N., Shin J.H., Sung H., Lee K., Kim E.C., Ryoo N., Lee J.S., Jung S.I., Park K.H., Kee S.J. (2009). Candida haemulonii and closely related species at 5 university hospitals in Korea: Identification, antifungal susceptibility, and clinical features. Clin. Infect. Dis..

[140] Lee W.G., Shin J.H., Uh Y., Kang M.G., Kim S.H., Park K.H., Jang H.C. (2011). First three reported cases of nosocomial fungemia caused by *Candida auris*. J. Clin. Microbiol..

[141] Forsberg K., Woodworth K., Walters M., Berkow E.L., Jackson B., Chiller T., Vallabhaneni S. (2019). *Candida auris*: The recent emergence of a multidrug-resistant fungal pathogen. Med. Mycol..

[142] Rhodes J., Fisher M.C. (2019). Global epidemiology of emerging *Candida auris*. Curr. Opin. Microbiol..

[143] Chow N.A., Gade L., Tsay S.V., Forsberg K., Greenko J.A., Southwick K.L., Barrett P.M., Kerins J.L., Lockhart S.R., Chiller T.M. (2018). Multiple introductions and subsequent transmission of multidrug-resistant *Candida auris* in the USA: A molecular epidemiological survey. Lancet Infect. Dis..

[144] Lockhart S.R., Etienne K.A., Vallabhaneni S., Farooqi J., Chowdhary A., Govender N.P., Colombo A.L., Calvo B., Cuomo C.A., Desjardins C.A. (2017). Simultaneous Emergence of Multidrug-Resistant *Candida auris* on 3 Continents Confirmed by Whole-Genome Sequencing and Epidemiological Analyses. Clin. Infect. Dis..

[145] Chayaporn S., Karrie Kwan Ki K., Kar Mun L., Mei Gie T., Patipan B., Joash Jun Keat C., Sui Sin G., Prevena R., Lai Chee L., Kwee Yuen T. (2023). Discovery of the sixth *Candida auris* clade in Singapore. medRxiv.

[146] Sharma C., Kumar N., Pandey R., Meis J.F., Chowdhary A. (2016). Whole genome sequencing of emerging multidrug resistant *Candida auris* isolates in India demonstrates low genetic variation. N. Microbes N. Infect..

[147] Tian S., Bing J., Chu Y., Chen J., Cheng S., Wang Q., Zhang J., Ma X., Zhou B., Liu L. (2021). Genomic epidemiology of *Candida auris* in a general hospital in Shenyang, China: A three-year surveillance study. Emerg. Microbes Infect..

[148] Tsay S., Welsh R.M., Adams E.H., Chow N.A., Gade L., Berkow E.L., Poirot E., Lutterloh E., Quinn M., Chaturvedi S. (2017). Notes from the Field: Ongoing Transmission of *Candida auris* in Health Care Facilities—United States, June 2016–May 2017. MMWR Morb. Mortal. Wkly. Rep..

[149] Piedrahita C.T., Cadnum J.L., Jencson A.L., Shaikh A.A., Ghannoum M.A., Donskey C.J. (2017). Environmental Surfaces in Healthcare Facilities are a Potential Source for Transmission of *Candida auris* and Other Candida Species. Infect. Control Hosp. Epidemiol..

[150] Larkin E., Hager C., Chandra J., Mukherjee P.K., Retuerto M., Salem I., Long L., Isham N., Kovanda L., Borroto-Esoda K. (2017). The Emerging Pathogen *Candida auris*: Growth Phenotype, Virulence Factors, Activity of Antifungals, and Effect of SCY-078, a Novel Glucan Synthesis Inhibitor, on Growth Morphology and Biofilm Formation. Antimicrob. Agents Chemother..

[151] Piatti G., Sartini M., Cusato C., Schito A.M. (2022). Colonization by *Candida auris* in critically ill patients: Role of cutaneous and rectal localization during an outbreak. J. Hosp. Infect..

[152] Chowdhary A., Anil Kumar V., Sharma C., Prakash A., Agarwal K., Babu R., Dinesh K.R., Karim S., Singh S.K., Hagen F. (2014). Multidrug-resistant endemic clonal strain of *Candida auris* in India. Eur. J. Clin. Microbiol. Infect. Dis..

[153] Rossow J., Ostrowsky B., Adams E., Greenko J., McDonald R., Vallabhaneni S., Forsberg K., Perez S., Lucas T., Alroy K.A. (2021). Factors Associated with *Candida auris* Colonization and Transmission in Skilled Nursing Facilities with Ventilator Units, New York, 2016–2018. Clin. Infect. Dis..

[154] Waters A., Chommanard C., Baltozer S., Angel L.C., Abdelfattah R., Lyman M., Forsberg K., Misas E., Litvintseva A.P., Fields V. (2023). Investigation of a *Candida auris* outbreak in a skilled nursing facility—Virginia, United States, October 2020–June 2021. Am. J. Infect. Control.

[155] Bergeron G., Bloch D., Murray K., Kratz M., Parton H., Ackelsberg J., Antwi M., Del Rosso P., Dorsinville M., Kubinson H. (2021). *Candida auris* Colonization After Discharge to a Community Setting: New York City, 2017–2019. Open Forum Infect. Dis..

[156] Patterson C.A., Wyncoll D., Patel A., Ceesay Y., Newsholme W., Chand M., Mitchell H., Tan M., Edgeworth J.D. (2021). Cloth Lanyards as a Source of Intermittent Transmission of *Candida auris* on an ICU∗. Crit. Care Med..

[157] Hinrichs C., Wiese-Posselt M., Graf B., Geffers C., Weikert B., Enghard P., Aldejohann A., Schrauder A., Knaust A., Eckardt K.U. (2022). Successful control of *Candida auris* transmission in a German COVID-19 intensive care unit. Mycoses.

[158] Sathyapalan D.T., Antony R., Nampoothiri V., Kumar A., Shashindran N., James J., Thomas J., Prasanna P., Sudhir A.S., Philip J.M. (2021). Evaluating the measures taken to contain a *Candida auris* outbreak in a tertiary care hospital in South India: An outbreak investigational study. BMC Infect. Dis..

[159] Modiri M., Khodavaisy S., Barac A., Akbari Dana M., Nazemi L., Aala F., Salehi M., Rezaie S. (2019). Comparison of biofilm-producing ability of clinical isolates of *Candida parapsilosis* species complex. J. Mycol. Med..

[160] Prigitano A., Perrone P.M., Esposto M.C., Carnevali D., De Nard F., Grimoldi L., Principi N., Cogliati M., Castaldi S., Romanò L. (2022). ICU environmental surfaces are a reservoir of fungi: Species distribution in northern Italy. J. Hosp. Infect..

[161] Zhang Z., Cao Y., Li Y., Chen X., Ding C., Liu Y. (2021). Risk factors and biofilm formation analyses of hospital-acquired infection of *Candida pelliculosa* in a neonatal intensive care unit. BMC Infect. Dis..

[162] Chowdhary A., Stielow J.B., Upadhyaya G., Singh P.K., Singh A., Meis J.F. (2020). *Candida blankii*: An emerging yeast in an outbreak of fungaemia in neonates in Delhi, India. Clin. Microbiol. Infect..

[163] Al-Sweih N., Ahmad S., Khan S., Joseph L., Asadzadeh M., Khan Z. (2019). *Cyberlindnera fabianii* fungaemia outbreak in preterm neonates in Kuwait and literature review. Mycoses.

[164] Xiao M., Wang H., Lu J., Chen S.C., Kong F., Ma X.J., Xu Y.C. (2014). Three clustered cases of candidemia caused by *Candida quercitrusa* and mycological characteristics of this novel species. J. Clin. Microbiol..

[165] Chakrabarti A., Singh K., Narang A., Singhi S., Batra R., Rao K.L., Ray P., Gopalan S., Das S., Gupta V. (2001). Outbreak of *Pichia anomala* infection in the pediatric service of a tertiary-care center in Northern India. J. Clin. Microbiol..

[166] Jung J., Moon Y.S., Yoo J.A., Lim J.H., Jeong J., Jun J.B. (2018). Investigation of a nosocomial outbreak of fungemia caused by *Candida pelliculosa* (*Pichia anomala*) in a Korean tertiary care center. J. Microbiol. Immunol. Infect..

[167] Kalenic S., Jandrlic M., Vegar V., Zuech N., Sekulic A., Mlinaric-Missoni E. (2001). Hansenula anomala outbreak at a surgical intensive care unit: A search for risk factors. Eur. J. Epidemiol..

[168] Pasqualotto A.C., Sukiennik T.C., Severo L.C., de Amorim C.S., Colombo A.L. (2005). An outbreak of *Pichia anomala* fungemia in a Brazilian pediatric intensive care unit. Infect. Control Hosp. Epidemiol..

[169] Rajendran R., Sherry L., Deshpande A., Johnson E.M., Hanson M.F., Williams C., Munro C.A., Jones B.L., Ramage G. (2016). A Prospective Surveillance Study of Candidaemia: Epidemiology, Risk Factors, Antifungal Treatment and Outcome in Hospitalized Patients. Front. Microbiol..

[170] Kalkanci A., Dizbay M., Turan O., Fidan I., Yalcin B., Hirfanoglu I., Kustimur S., Aktas F., Sugita T. (2010). Nosocomial transmission of *Candida pelliculosa* fungemia in a pediatric intensive care unit and review of the literature. Turk. J. Pediatr..

[171] Lin H.C., Lin H.Y., Su B.H., Ho M.W., Ho C.M., Lee C.Y., Lin M.H., Hsieh H.Y., Lin H.C., Li T.C. (2013). Reporting an outbreak of *Candida pelliculosa* fungemia in a neonatal intensive care unit. J. Microbiol. Immunol. Infect..

[172] Spruijtenburg B., Rudramurthy S.M., Meijer E.F.J., van Haren M.H.I., Kaur H., Chakrabarti A., Meis J.F., de Groot T. (2023). Application of Novel Short Tandem Repeat Typing for Wickerhamomyces anomalus Reveals Simultaneous Outbreaks within a Single Hospital. Microorganisms.

[173] Fan X., Dai R.C., Kudinha T., Gu L. (2023). A pseudo-outbreak of *Cyberlindnera fabianii* funguria: Implication from whole genome sequencing assay. Front. Cell. Infect. Microbiol..

[174] Menu E., Criscuolo A., Desnos-Ollivier M., Cassagne C., D’Incan E., Furst S., Ranque S., Berger P., Dromer F. (2020). Saprochaete clavata Outbreak Infecting Cancer Center through Dishwasher. Emerg. Infect. Dis..

[175] Vaux S., Criscuolo A., Desnos-Ollivier M., Diancourt L., Tarnaud C., Vandenbogaert M., Brisse S., Coignard B., Dromer F., Geotrichum Investigation G. (2014). Multicenter outbreak of infections by Saprochaete clavata, an unrecognized opportunistic fungal pathogen. mBio.

[176] Lo Cascio G., Vincenzi M., Soldani F., De Carolis E., Maccacaro L., Sorrentino A., Nadali G., Cesaro S., Sommavilla M., Niero V. (2020). Outbreak of Saprochaete clavata Sepsis in Hematology Patients: Combined Use of MALDI-TOF and Sequencing Strategy to Identify and Correlate the Episodes. Front. Microbiol..

[177] Stanzani M., Cricca M., Sassi C., Sutto E., De Cicco G., Bonifazi F., Bertuzzi C., Bacci F., Paolini S., Cavo M. (2019). Saprochaete clavata infections in patients undergoing treatment for haematological malignancies: A report of a monocentric outbreak and review of the literature. Mycoses.

[178] Huang J.J., Chen X.F., Tsui C.K.M., Pang C.J., Hu Z.D., Shi Y., Wang W.P., Cui L.Y., Xiao Y.L., Gong J. (2022). Persistence of an epidemic cluster of Rhodotorula mucilaginosa in multiple geographic regions in China and the emergence of a 5-flucytosine resistant clone. Emerg. Microbes Infect..

[179] Keighley C., Chen S.C., Marriott D., Pope A., Chapman B., Kennedy K., Bak N., Underwood N., Wilson H.L., McDonald K. (2019). Candidaemia and a risk predictive model for overall mortality: A prospective multicentre study. BMC Infect. Dis..

[180] Engelthaler D.M., Hicks N.D., Gillece J.D., Roe C.C., Schupp J.M., Driebe E.M., Gilgado F., Carriconde F., Trilles L., Firacative C. (2014). *Cryptococcus gattii* in North American Pacific Northwest: Whole-population genome analysis provides insights into species evolution and dispersal. mBio.

[181] Gerstein A.C., Jackson K.M., McDonald T.R., Wang Y., Lueck B.D., Bohjanen S., Smith K.D., Akampurira A., Meya D.B., Xue C. (2019). Identification of Pathogen Genomic Differences That Impact Human Immune Response and Disease during *Cryptococcus neoformans* Infection. mBio.

[182] Meyer W. (2015). *Cryptococcus gattii* in the Age of Whole-Genome Sequencing. mBio.

[183] Patino L.H., Munoz M., Ramirez A.L., Velez N., Escandon P., Parra-Giraldo C.M., Ramirez J.D. (2023). A Landscape of the Genomic Structure of *Cryptococcus neoformans* in Colombian Isolates. J. Fungi.

[184] Farrer R.A., Borman A.M., Inkster T., Fisher M.C., Johnson E.M., Cuomo C.A. (2021). Genomic epidemiology of a *Cryptococcus neoformans* case cluster in Glasgow, Scotland, 2018. Microb. Genom..

[185] Miozzo I., Aquino V.R., Duarte M., Santos R.P., Goldani L.Z. (2010). *Cryptococcus neoformans* as a rare cause of hospital infection. Infect. Control Hosp. Epidemiol..

[186] Wang C.Y., Wu H.D., Hsueh P.R. (2005). Nosocomial transmission of cryptococcosis. N. Engl. J. Med..

[187] Glaser J.B., Garden A. (1985). Inoculation of cryptococcosis without transmission of the acquired immunodeficiency syndrome. N. Engl. J. Med..

[188] Kaul D.R., Vece G., Blumberg E., La Hoz R.M., Ison M.G., Green M., Pruett T., Nalesnik M.A., Tlusty S.M., Wilk A.R. (2021). Ten years of donor-derived disease: A report of the disease transmission advisory committee. Am. J. Transplant..

[189] Natarajan P., Lockhart S.R., Basavaraju S.V., Anjan S., Lindsley M.D., McGrath M.M., Oh D.H., Jackson B.R. (2021). Donor-derived *Cryptococcus gattii* sensu stricto infection in two kidney transplant recipients, southeastern United States. Am. J. Transplant..

[190] Kennedy E., Vanichanan J., Rajapreyar I., Gonzalez B., Nathan S., Gregoric I., Kar B., Loyalka P., Weeks P., Chavez V. (2016). A pseudo-outbreak of disseminated cryptococcal disease after orthotopic heart transplantation. Mycoses.

[191] Vallabhaneni S., Haselow D., Lloyd S., Lockhart S., Moulton-Meissner H., Lester L., Wheeler G., Gladden L., Garner K., Derado G. (2015). Cluster of *Cryptococcus neoformans* Infections in Intensive Care Unit, Arkansas, USA, 2013. Emerg. Infect. Dis..

[192] Benedict K., Mody R.K. (2016). Epidemiology of Histoplasmosis Outbreaks, United States, 1938–2013. Emerg. Infect. Dis..

[193] Freedman M., Jackson B.R., McCotter O., Benedict K. (2018). Coccidioidomycosis Outbreaks, United States and Worldwide, 1940–2015. Emerg. Infect. Dis..

[194] Thompson G.R., Le T., Chindamporn A., Kauffman C.A., Alastruey-Izquierdo A., Ampel N.M., Andes D.R., Armstrong-James D., Ayanlowo O., Baddley J.W. (2021). Global guideline for the diagnosis and management of the endemic mycoses: An initiative of the European Confederation of Medical Mycology in cooperation with the International Society for Human and Animal Mycology. Lancet Infect. Dis..

[195] Engelthaler D.M., Chiller T., Schupp J.A., Colvin J., Beckstrom-Sternberg S.M., Driebe E.M., Moses T., Tembe W., Sinari S., Beckstrom-Sternberg J.S. (2011). Next-generation sequencing of Coccidioides immitis isolated during cluster investigation. Emerg. Infect. Dis..

[196] Jewell K., Cheshier R., Cage G.D. (2008). Genetic diversity among clinical Coccidioides spp. isolates in Arizona. Med. Mycol..

[197] Alangaden G.J. (2011). Nosocomial fungal infections: Epidemiology, infection control, and prevention. Infect. Dis. Clin. N. Am..

[198] Delliere S., Gits-Muselli M., Bretagne S., Alanio A. (2020). Outbreak-Causing Fungi: *Pneumocystis jirovecii*. Mycopathologia.

[199] Le Gal S., Toubas D., Totet A., Dalle F., Abou Bacar A., Le Meur Y., Nevez G., Anofel A. (2020). Pneumocystis Infection Outbreaks in Organ Transplantation Units in France: A Nation-Wide Survey. Clin. Infect. Dis..

[200] Yiannakis E.P., Boswell T.C. (2016). Systematic review of outbreaks of *Pneumocystis jirovecii* pneumonia: Evidence that *P. jirovecii* is a transmissible organism and the implications for healthcare infection control. J. Hosp. Infect..

[201] Miller R.F., Ambrose H.E., Wakefield A.E. (2001). *Pneumocystis carinii* f. sp. hominis DNA in immunocompetent health care workers in contact with patients with *P. carinii* pneumonia. J. Clin. Microbiol..

[202] Valade S., Azoulay E., Damiani C., Derouin F., Totet A., Menotti J. (2015). *Pneumocystis jirovecii* airborne transmission between critically ill patients and health care workers. Intensive Care Med..

[203] Wakefield A.E. (1996). DNA sequences identical to *Pneumocystis carinii* f. s*P. carinii* and *Pneumocystis carinii* f. sp. hominis in samples of air spora. J. Clin. Microbiol..

[204] Morris A., Norris K.A. (2012). Colonization by *Pneumocystis jirovecii* and its role in disease. Clin. Microbiol. Rev..

[205] Alanio A., Gits-Muselli M., Guigue N., Desnos-Ollivier M., Calderon E.J., Di Cave D., Dupont D., Hamprecht A., Hauser P.M., Helweg-Larsen J. (2017). Diversity of *Pneumocystis jirovecii* Across Europe: A Multicentre Observational Study. EBioMedicine.

[206] Brunot V., Pernin V., Chartier C., Garrigue V., Vetromile F., Szwarc I., Delmas S., Portales P., Basset D., Mourad G. (2012). An epidemic of *Pneumocystis jiroveci* pneumonia in a renal transplantation center: Role of T-cell lymphopenia. Transplant. Proc..

[207] Gianella S., Haeberli L., Joos B., Ledergerber B., Wuthrich R.P., Weber R., Kuster H., Hauser P.M., Fehr T., Mueller N.J. (2010). Molecular evidence of interhuman transmission in an outbreak of *Pneumocystis jirovecii* pneumonia among renal transplant recipients. Transpl. Infect. Dis..

[208] Mulpuru S., Knoll G., Weir C., Desjardins M., Johnson D., Gorn I., Fairhead T., Bissonnette J., Bruce N., Toye B. (2016). Pneumocystis pneumonia outbreak among renal transplant recipients at a North American transplant center: Risk factors and implications for infection control. Am. J. Infect. Control.

[209] Desoubeaux G., Dominique M., Morio F., Thepault R.A., Franck-Martel C., Tellier A.C., Ferrandiere M., Hennequin C., Bernard L., Salame E. (2016). Epidemiological Outbreaks of *Pneumocystis jirovecii* Pneumonia Are Not Limited to Kidney Transplant Recipients: Genotyping Confirms Common Source of Transmission in a Liver Transplantation Unit. J. Clin. Microbiol..

[210] Miguel Montanes R., Elkrief L., Hajage D., Houssel P., Fantin B., Francoz C., Dreyfuss D., Ricard J.D., Durand F. (2018). An outbreak of Pneumocytis jirovecii pneumonia among liver transplant recipients. Transpl. Infect. Dis..

[211] Rostved A.A., Sassi M., Kurtzhals J.A., Sorensen S.S., Rasmussen A., Ross C., Gogineni E., Huber C., Kutty G., Kovacs J.A. (2013). Outbreak of pneumocystis pneumonia in renal and liver transplant patients caused by genotypically distinct strains of *Pneumocystis jirovecii*. Transplantation.

[212] Olsson M., Eriksson B.M., Elvin K., Strandberg M., Wahlgren M. (2001). Genotypes of clustered cases of *Pneumocystis carinii* pneumonia. Scand. J. Infect. Dis..

[213] Mori S., Sugimoto M. (2012). *Pneumocystis jirovecii* infection: An emerging threat to patients with rheumatoid arthritis. Rheumatology.

[214] de Boer M.G., de Fijter J.W., Kroon F.P. (2011). Outbreaks and clustering of Pneumocystis pneumonia in kidney transplant recipients: A systematic review. Med. Mycol..

[215] de Boer M.G., Bruijnesteijn van Coppenraet L.E., Gaasbeek A., Berger S.P., Gelinck L.B., van Houwelingen H.C., van den Broek P., Kuijper E.J., Kroon F.P., Vandenbroucke J.P. (2007). An outbreak of *Pneumocystis jiroveci* pneumonia with 1 predominant genotype among renal transplant recipients: Interhuman transmission or a common environmental source?. Clin. Infect. Dis..

[216] Nevez G., Le Gal S., Noel N., Wynckel A., Huguenin A., Le Govic Y., Pougnet L., Virmaux M., Toubas D., Bajolet O. (2018). Investigation of nosocomial pneumocystis infections: Usefulness of longitudinal screening of epidemic and post-epidemic pneumocystis genotypes. J. Hosp. Infect..

[217] Maitte C., Leterrier M., Le Pape P., Miegeville M., Morio F. (2013). Multilocus sequence typing of *Pneumocystis jirovecii* from clinical samples: How many and which loci should be used?. J. Clin. Microbiol..

[218] Urabe N., Ishii Y., Hyodo Y., Aoki K., Yoshizawa S., Saga T., Murayama S.Y., Sakai K., Homma S., Tateda K. (2016). Molecular epidemiologic analysis of a Pneumocystis pneumonia outbreak among renal transplant patients. Clin. Microbiol. Infect..

[219] Charpentier E., Garnaud C., Wintenberger C., Bailly S., Murat J.B., Rendu J., Pavese P., Drouet T., Augier C., Malvezzi P. (2017). Added Value of Next-Generation Sequencing for Multilocus Sequence Typing Analysis of a *Pneumocystis jirovecii* Pneumonia Outbreak1. Emerg. Infect. Dis..

[220] Kanamori H., Rutala W.A., Sickbert-Bennett E.E., Weber D.J. (2015). Review of fungal outbreaks and infection prevention in healthcare settings during construction and renovation. Clin. Infect. Dis..

[221] Mareković I. (2023). What’s New in Prevention of Invasive Fungal Diseases during Hospital Construction and Renovation Work: An Overview. J. Fungi.

[222] Vonberg R.P., Gastmeier P. (2006). Nosocomial aspergillosis in outbreak settings. J. Hosp. Infect..

[223] Pini G., Faggi E., Donato R., Sacco C., Fanci R. (2008). Invasive pulmonary aspergillosis in neutropenic patients and the influence of hospital renovation. Mycoses.

[224] Loo V.G., Bertrand C., Dixon C., Vityé D., DeSalis B., McLean A.P., Brox A., Robson H.G. (1996). Control of construction-associated nosocomial aspergillosis in an antiquated hematology unit. Infect. Control Hosp. Epidemiol..

[225] Anderson K., Morris G., Kennedy H., Croall J., Michie J., Richardson M.D., Gibson B. (1996). Aspergillosis in immunocompromised paediatric patients: Associations with building hygiene, design, and indoor air. Thorax.

[226] Lee L.D., Hachem R.Y., Berkheiser M., Hackett B., Jiang Y., Raad I.I. (2012). Hospital environment and invasive aspergillosis in patients with hematologic malignancy. Am. J. Infect. Control.

[227] Lai K.K. (2001). A cluster of invasive aspergillosis in a bone marrow transplant unit related to construction and the utility of air sampling. Am. J. Infect. Control.

[228] Chang C.C., Cheng A.C., Devitt B., Hughes A.J., Campbell P., Styles K., Low J., Athan E. (2008). Successful control of an outbreak of invasive aspergillosis in a regional haematology unit during hospital construction works. J. Hosp. Infect..

[229] Thio C.L., Smith D., Merz W.G., Streifel A.J., Bova G., Gay L., Miller C.B., Perl T.M. (2000). Refinements of environmental assessment during an outbreak investigation of invasive aspergillosis in a leukemia and bone marrow transplant unit. Infect. Control Hosp. Epidemiol..

[230] Hahn T., Cummings K.M., Michalek A.M., Lipman B.J., Segal B.H., McCarthy P.L. (2002). Efficacy of high-efficiency particulate air filtration in preventing aspergillosis in immunocompromised patients with hematologic malignancies. Infect. Control Hosp. Epidemiol..

[231] Panackal A.A., Dahlman A., Keil K.T., Peterson C.L., Mascola L., Mirza S., Phelan M., Lasker B.A., Brandt M.E., Carpenter J. (2003). Outbreak of invasive aspergillosis among renal transplant recipients. Transplantation.

[232] Lee L.D., Berkheiser M., Jiang Y., Hackett B., Hachem R.Y., Chemaly R.F., Raad I.I. (2007). Risk of bioaerosol contamination with *Aspergillus* species before and after cleaning in rooms filtered with high-efficiency particulate air filters that house patients with hematologic malignancy. Infect. Control Hosp. Epidemiol..

[233] Richardson M., Rautemaa-Richardson R. (2019). Exposure to *Aspergillus* in Home and Healthcare Facilities’ Water Environments: Focus on Biofilms. Microorganisms.

[234] Babic M.N., Gunde-Cimerman N., Vargha M., Tischner Z., Magyar M., Verissimo C., Sabino R., Viegas C., Meyer W., Brandao J. (2017). Fungal Contaminants in Drinking Water Regulation? A Tale of Ecology, Exposure, Purification and Clinical Relevance. Int. J. Environ. Res. Public Health.

[235] Moat J., Rizoulis A., Fox G., Upton M. (2016). Domestic shower hose biofilms contain fungal species capable of causing opportunistic infection. J. Water Health.

[236] Oliveira L.T., Lopes L.G., Ramos S.B., Martins C.H.G., Jamur M.C., Pires R.H. (2018). Fungal biofilms in the hemodialysis environment. Microb. Pathog..

[237] Gonzalez-Ramirez A.I., Ramirez-Granillo A., Medina-Canales M.G., Rodriguez-Tovar A.V., Martinez-Rivera M.A. (2016). Analysis and description of the stages of *Aspergillus* fumigatus biofilm formation using scanning electron microscopy. BMC Microbiol..

[238] Kauffmann-Lacroix C., Costa D., Imbert C. (2016). Fungi, Water Supply and Biofilms. Adv. Exp. Med. Biol..

[239] Vanhee L.M., Symoens F., Jacobsen M.D., Nelis H.J., Coenye T. (2009). Comparison of multiple typing methods for *Aspergillus* fumigatus. Clin. Microbiol. Infect..

[240] Ao J.H., Hao Z.F., Zhu H., Wen L., Yang R.Y. (2014). Environmental investigations and molecular typing of *Aspergillus* in a Chinese hospital. Mycopathologia.

[241] Misas E., Deng J.Z., Gold J.A.W., Gade L., Nunnally N.S., Georgacopoulos O., Bentz M., Berkow E.L., Litvintseva A.P., Chiller T.M. (2023). Genomic description of human clinical *Aspergillus* fumigatus isolates, California, 2020. Med. Mycol..

[242] Gheith S., Saghrouni F., Normand A.C., Bannour W., Khelif A., Piarroux R., Ben Said M., Njah M., Ranque S. (2016). Microsatellite Typing of *Aspergillus* flavus Strains in a Tunisian Onco-hematology Unit. Mycopathologia.

[243] Heinemann S., Symoens F., Gordts B., Jannes H., Nolard N. (2004). Environmental investigations and molecular typing of *Aspergillus* flavus during an outbreak of postoperative infections. J. Hosp. Infect..

[244] Loeffert S.T., Melloul E., Gustin M.P., Henaff L., Guillot C., Dupont D., Wallon M., Cassier P., Dananche C., Benet T. (2019). Investigation of the Relationships Between Clinical and Environmental Isolates of *Aspergillus* fumigatus by Multiple-locus Variable Number Tandem Repeat Analysis during Major Demolition Work in a French Hospital. Clin. Infect. Dis..

[245] Kidd S.E., Ling L.M., Meyer W., Orla Morrissey C., Chen S.C., Slavin M.A. (2009). Molecular epidemiology of invasive aspergillosis: Lessons learned from an outbreak investigation in an Australian hematology unit. Infect. Control Hosp. Epidemiol..

[246] Cortez K.J., Roilides E., Quiroz-Telles F., Meletiadis J., Antachopoulos C., Knudsen T., Buchanan W., Milanovich J., Sutton D.A., Fothergill A. (2008). Infections caused by *Scedosporium* spp. Clin. Microbiol. Rev..

[247] Ramirez-Garcia A., Pellon A., Rementeria A., Buldain I., Barreto-Bergter E., Rollin-Pinheiro R., de Meirelles J.V., Xisto M., Ranque S., Havlicek V. (2018). *Scedosporium* and *Lomentospora*: An updated overview of underrated opportunists. Med. Mycol..

[248] Guerrero A., Torres P., Duran M.T., Ruiz-Díez B., Rosales M., Rodriguez-Tudela J.L. (2001). Airborne outbreak of nosocomial *Scedosporium prolificans* infection. Lancet.

[249] Alvarez M., Lopez Ponga B., Rayon C., Garcia Gala J., Roson Porto M.C., Gonzalez M., Martinez-Suarez J.V., Rodriguez-Tudela J.L. (1995). Nosocomial outbreak caused by *Scedosporium prolificans* (inflatum): Four fatal cases in leukemic patients. J. Clin. Microbiol..

[250] Ruiz-Díez B., Martín-Díez F., Rodríguez-Tudela J.L., Alvárez M., Martínez-Suárez J.V. (1997). Use of random amplification of polymorphic DNA (RAPD) and PCR-fingerprinting for genotyping a *Scedosporium prolificans* (inflatum) outbreak in four leukemic patients. Curr. Microbiol..

[251] Boan P., Pang S., Gardam D.J., Darragh H., Wright M., Coombs G.W. (2020). Investigation of a *Lomentospora* prolificans case cluster with whole genome sequencing. Med. Mycol. Case Rep..

[252] Bernhardt A., Seibold M., Rickerts V., Tintelnot K. (2015). Cluster analysis of *Scedosporium* boydii infections in a single hospital. Int. J. Med. Microbiol..

[253] Carlesse F., Amaral A.C., Gonçalves S.S., Xafranski H., Lee M.M., Zecchin V., Petrilli A.S., Al-Hatmi A.M., Hagen F., Meis J.F. (2017). Outbreak of *Fusarium oxysporum* infections in children with cancer: An experience with 7 episodes of catheter-related fungemia. Antimicrob. Resist. Infect. Control.

[254] Yoon S.J., Kim S.H., Bahk H.J., Ahn Y.S., Lee J.J., Kim H.J., Lim H.J., Choi M.J., Shin J.H., Lee Y.K. (2022). Fungal Endophthalmitis Outbreak after Cataract Surgery, South Korea, 2020. Emerg. Infect. Dis..

[255] Rickerts V., Bohme A., Viertel A., Behrendt G., Jacobi V., Tintelnot K., Just-Nubling G. (2000). Cluster of pulmonary infections caused by *Cunninghamella bertholletiae* in immunocompromised patients. Clin. Infect. Dis..

[256] Verhasselt H.L., Radke J., Schmidt D., Killengray D., Scharmann U., Rickerts V., Hansen W., Seidel D., Falces-Romero I., Buer J. (2019). Comparison of genotyping methods for *Cunninghamella bertholletiae*. Mycoses.

[257] Garcia-Hermoso D., Criscuolo A., Lee S.C., Legrand M., Chaouat M., Denis B., Lafaurie M., Rouveau M., Soler C., Schaal J.V. (2018). Outbreak of Invasive Wound Mucormycosis in a Burn Unit Due to Multiple Strains of *Mucor circinelloides* f. *circinelloides* Resolved by Whole-Genome Sequencing. mBio.

[258] Gamarra S., Chaves M.S., Cabeza M.S., Macedo D., Leonardelli F., Franco D., Boleas M., Garcia-Effron G. (2018). Mucormycosis outbreak due to *Rhizopus microsporus* after arthroscopic anterior cruciate ligament reconstruction surgery evaluated by RAPD and MALDI-TOF Mass spectrometry. J. Mycol. Med..

[259] Marek C., Croxen M.A., Dingle T.C., Bharat A., Schwartz I.S., Wiens R., Smith S. (2019). The use of genome sequencing to investigate an outbreak of hospital-acquired mucormycosis in transplant patients. Transpl. Infect. Dis..

[260] Nguyen M.H., Kaul D., Muto C., Cheng S.J., Richter R.A., Bruno V.M., Liu G., Beyhan S., Sundermann A.J., Mounaud S. (2020). Genetic diversity of clinical and environmental Mucorales isolates obtained from an investigation of mucormycosis cases among solid organ transplant recipients. Microb. Genom..

[261] Litvintseva A.P., Hurst S., Gade L., Frace M.A., Hilsabeck R., Schupp J.M., Gillece J.D., Roe C., Smith D., Keim P. (2014). Whole-genome analysis of Exserohilum rostratum from an outbreak of fungal meningitis and other infections. J. Clin. Microbiol..

[262] Etienne K.A., Roe C.C., Smith R.M., Vallabhaneni S., Duarte C., Escadon P., Castaneda E., Gomez B.L., de Bedout C., Lopez L.F. (2016). Whole-Genome Sequencing to Determine Origin of Multinational Outbreak of Sarocladium kiliense Bloodstream Infections. Emerg. Infect. Dis..

[263] Bupha-Intr O., Butters C., Reynolds G., Kennedy K., Meyer W., Patil S., Bryant P., Morrissey C.O., Australasian Antifungal Guidelines Steering Committee (2021). Consensus guidelines for the diagnosis and management of invasive fungal disease due to moulds other than *Aspergillus* in the haematology/oncology setting, 2021. Intern. Med. J..

[264] Buchta V., Feuermannová A., Váša M., Bašková L., Kutová R., Kubátová A., Vejsová M. (2014). Outbreak of fungal endophthalmitis due to *Fusarium oxysporum* following cataract surgery. Mycopathologia.

[265] Mukherjee P.K., Chandra J., Yu C., Sun Y., Pearlman E., Ghannoum M.A. (2012). Characterization of *Fusarium* keratitis outbreak isolates: Contribution of biofilms to antimicrobial resistance and pathogenesis. Invest. Ophthalmol. Vis. Sci..

[266] Levy B., Heiler D., Norton S. (2006). Report on testing from an investigation of *Fusarium* keratitis in contact lens wearers. Eye Contact Lens.

[267] Sugar J. (2006). The contact lens/*Fusarium* “outbreak”. Am. J. Ophthalmol..

[268] Chang D.C., Grant G.B., O’Donnell K., Wannemuehler K.A., Noble-Wang J., Rao C.Y., Jacobson L.M., Crowell C.S., Sneed R.S., Lewis F.M. (2006). Multistate outbreak of *Fusarium* keratitis associated with use of a contact lens solution. JAMA.

[269] Arasaki R., Tanaka S., Okawa K., Tanaka Y., Inoue T., Kobayashi S., Ito A., Maruyama-Inoue M., Yamaguchi T., Muraosa Y. (2022). Endophthalmitis outbreak caused by *Fusarium oxysporum* after cataract surgery. Am. J. Ophthalmol. Case Rep..

[270] Cakir M., Imamoğlu S., Cekiç O., Bozkurt E., Alagöz N., Oksüz L., Yilmaz O.F. (2009). An outbreak of early-onset endophthalmitis caused by *Fusarium* species following cataract surgery. Curr. Eye Res..

[271] Alagöz N. (2020). Ten years after an outbreak of *Fusarium* endophthalmitis following cataract surgery. Arq. Bras. Oftalmol..

[272] Kim S.W., Kim J.H., Choi M., Lee S.J., Shin J.P., Kim J.G., Kang S.W., Park K.H. (2023). An Outbreak of Fungal Endophthalmitis After Cataract Surgery in South Korea. JAMA Ophthalmol..

[273] Güngel H., Eren M.H., Pınarcı E.Y., Altan C., Baylançiçek D.O., Kara N., Gürsel T., Yegenoğlu Y., Susever S. (2011). An outbreak of *Fusarium solani* endophthalmitis after cataract surgery in an eye training and research hospital in Istanbul. Mycoses.

[274] Santonato D., Novau A., Fabbro L., Paulosky L., Panez S., Pineda G., Prado F., Rivas M.M., Sevilla S., Pereyra M.L. (2020). A set of environmental measures to control a *Fusarium* outbreak in an oncohematologic ward: An interrupted time series study. Infect. Control Hosp. Epidemiol..

[275] Litvinov N., da Silva M.T., van der Heijden I.M., Graça M.G., Marques de Oliveira L., Fu L., Giudice M., Zilda de Aquino M., Odone-Filho V., Marques H.H. (2015). An outbreak of invasive fusariosis in a children’s cancer hospital. Clin. Microbiol. Infect..

[276] Mohorea-Neata A.L., Ghita M.C., Moroti R., Ghiaur A., Ionescu B., Tatic A., Stancioaica M.C., Bardas A., Al-Hatmi A., Coriu D. (2023). Invasive fusariosis in acute leukaemia patients—An outbreak in the haematology ward. Mycoses.

[277] Georgiadou S.P., Velegraki A., Arabatzis M., Neonakis I., Chatzipanagiotou S., Dalekos G.N., Petinaki E. (2014). Cluster of *Fusarium verticillioides* bloodstream infections among immunocompetent patients in an internal medicine department after reconstruction works in Larissa, Central Greece. J. Hosp. Infect..

[278] Centers for Disease Control and Prevention Fungal Meningitis Outbreak Associated with Procedures Performed under Epidural Anesthesia in Matamoros, Mexico. https://www.cdc.gov/hai/outbreaks/meningitis-epidural-anesthesia.html.

[279] Smith D.J., Gold J.A.W., Chiller T., Bustamante N.D., Marinissen M.J., Rodriquez G.G., Cortes V.B.G., Molina C.D., Williams S., Vazquez Deida A.A. (2023). Update on Outbreak of Fungal Meningitis among U.S. Residents who Received Epidural Anesthesia at Two Clinics in Matamoros, Mexico. Clin. Infect. Dis..

[280] Baum S.G. NEJM Journal Watch: *Fusarium* Meningitis as a Consequence of Medical Tourism. https://www.jwatch.org/na56257/2023/06/23/fusarium-meningitis-consequence-medical-tourism.

[281] Sundermann A.J., Clancy C.J., Pasculle A.W., Liu G., Cheng S., Cumbie R.B., Driscoll E., Ayres A., Donahue L., Buck M. (2022). Remediation of Mucorales-contaminated Healthcare Linens at a Laundry Facility Following an Investigation of a Case Cluster of Hospital-acquired Mucormycosis. Clin. Infect. Dis..

[282] Davoudi S., Graviss L.S., Kontoyiannis D.P. (2015). Healthcare-associated outbreaks due to Mucorales and other uncommon fungi. Eur. J. Clin. Investig..

[283] Walther G., Wagner L., Kurzai O. (2020). Outbreaks of Mucorales and the Species Involved. Mycopathologia.

[284] Cheng V.C.C., Chen J.H.K., Wong S.C.Y., Leung S.S.M., So S.Y.C., Lung D.C., Lee W.M., Trendell-Smith N.J., Chan W.M., Ng D. (2016). Hospital Outbreak of Pulmonary and Cutaneous Zygomycosis due to Contaminated Linen Items from Substandard Laundry. Clin. Infect. Dis..

[285] Teal L.J., Schultz K.M., Weber D.J., Gergen M.F., Miller M.B., DiBiase L.M., Sickbert-Bennett E.E., Rutala W.A. (2016). Invasive Cutaneous Rhizopus Infections in an Immunocompromised Patient Population Associated with Hospital Laundry Carts. Infect. Control Hosp. Epidemiol..

[286] Duffy J., Harris J., Gade L., Sehulster L., Newhouse E., O’Connell H., Noble-Wang J., Rao C., Balajee S.A., Chiller T. (2014). Mucormycosis outbreak associated with hospital linens. Pediatr. Infect. Dis. J..

[287] Sundermann A.J., Clancy C.J., Pasculle A.W., Liu G., Cumbie R.B., Driscoll E., Ayres A., Donahue L., Pergam S.A., Abbo L. (2019). How Clean Is the Linen at My Hospital? The Mucorales on Unclean Linen Discovery Study of Large United States Transplant and Cancer Centers. Clin. Infect. Dis..

[288] Biswal M., Gupta P., Kanaujia R., Kaur K., Kaur H., Vyas A., Hallur V., Behera B., Padaki P., Savio J. (2022). Evaluation of hospital environment for presence of Mucorales during COVID-19-associated mucormycosis outbreak in India—A multi-centre study. J. Hosp. Infect..

[289] Ritter J.M., Muehlenbachs A., Blau D.M., Paddock C.D., Shieh W.J., Drew C.P., Batten B.C., Bartlett J.H., Metcalfe M.G., Pham C.D. (2013). Exserohilum infections associated with contaminated steroid injections: A clinicopathologic review of 40 cases. Am. J. Pathol..

[290] (2012). Multistate outbreak of fungal infection associated with injection of methylprednisolone acetate solution from a single compounding pharmacy—United States, 2012. MMWR Morb. Mortal. Wkly. Rep..

[291] Smith R.M., Schaefer M.K., Kainer M.A., Wise M., Finks J., Duwve J., Fontaine E., Chu A., Carothers B., Reilly A. (2013). Fungal infections associated with contaminated methylprednisolone injections. N. Engl. J. Med..

[292] Orsini M., Otaíza F., Vega P., Hederra L.M., Pidal P., Salas V., Coria P., Urízar C., Sepúlveda D., Palma M. (2018). Outbreak of fungemias by Sarocladium kiliense in eight public hospitals per intrinsic contamination of ondansetron intravenous. Rev. Chil. Infectol..

[293] Hartnett K.P., Jackson B.R., Perkins K.M., Glowicz J., Kerins J.L., Black S.R., Lockhart S.R., Christensen B.E., Beer K.D. (2019). A Guide to Investigating Suspected Outbreaks of Mucormycosis in Healthcare. J. Fungi.

[294] Nicolle M.C., Benet T., Vanhems P. (2011). Aspergillosis: Nosocomial or community-acquired?. Med. Mycol..

[295] Nawada R., Amitani R., Tanaka E., Niimi A., Suzuki K., Murayama T., Kuze F. (1996). Murine model of invasive pulmonary aspergillosis following an earlier stage, noninvasive *Aspergillus* infection. J. Clin. Microbiol..

[296] Benet T., Voirin N., Nicolle M.C., Picot S., Michallet M., Vanhems P. (2013). Estimation of the incubation period of invasive aspergillosis by survival models in acute myeloid leukemia patients. Med. Mycol..

[297] Smith R.M., Lee J., Mody R.K. (2015). Determining the Incubation Time of Mucormycosis: A Systematic Review. Open Forum Infect. Dis..

[298] Neblett Fanfair R., Benedict K., Bos J., Bennett S.D., Lo Y.C., Adebanjo T., Etienne K., Deak E., Derado G., Shieh W.J. (2012). Necrotizing cutaneous mucormycosis after a tornado in Joplin, Missouri, in 2011. N. Engl. J. Med..

[299] Khanina A., Tio S.Y., Ananda-Rajah M.R., Kidd S.E., Williams E., Chee L., Urbancic K., Thursky K.A. (2021). Consensus guidelines for antifungal stewardship, surveillance and infection prevention, 2021. Intern. Med. J..

[300] Baggio D., Peel T., Peleg A.Y., Avery S., Prayaga M., Foo M., Haffari G., Liu M., Bergmeir C., Ananda-Rajah M. (2019). Closing the Gap in Surveillance and Audit of Invasive Mold Diseases for Antifungal Stewardship Using Machine Learning. J. Clin. Med..

[301] Chang C.C., Ananda-Rajah M., Belcastro A., McMullan B., Reid A., Dempsey K., Athan E., Cheng A.C., Slavin M.A. (2014). Consensus guidelines for implementation of quality processes to prevent invasive fungal disease and enhanced surveillance measures during hospital building works, 2014. Intern. Med. J..

[302] Ong C.W., Chen S.C., Clark J.E., Halliday C.L., Kidd S.E., Marriott D.J., Marshall C.L., Morris A.J., Morrissey C.O., Roy R. (2019). Diagnosis, management and prevention of *Candida auris* in hospitals: Position statement of the Australasian Society for Infectious Diseases. Intern. Med. J..

[303] European Centre for Disease Prevention and Control *Candida auris* in Healthcare Settings—Europe. https://www.ecdc.europa.eu/sites/default/files/documents/RRA-Candida-auris-European-Union-countries.pdf.

[304] Centers of Disease Control and Prevention Infection Prevention and Control for *Candida auris*. https://www.cdc.gov/fungal/candida-auris/c-auris-infection-control.html.

[305] Public Health England Guidance for the Laboratory Investigation, Management and Infection Prevention and Control for Cases of *Candida auris* August 2017 v2.0. https://assets.publishing.service.gov.uk/government/uploads/system/uploads/attachment_data/file/637685/Updated_Candida_auris_Guidance_v2.pdf.

[306] Centers for Disease Control and Prevention Implementation of Personal Protective Equipment (PPE) Use in Nursing Homes to Prevent Spread of Multidrug-Resistant Organisms (MDROs). https://www.cdc.gov/hai/containment/PPE-Nursing-Homes.html.

[307] Pacilli M., Kerins J.L., Clegg W.J., Walblay K.A., Adil H., Kemble S.K., Xydis S., McPherson T.D., Lin M.Y., Hayden M.K. (2020). Regional Emergence of *Candida auris* in Chicago and Lessons Learned From Intensive Follow-up at 1 Ventilator-Capable Skilled Nursing Facility. Clin. Infect. Dis..

[308] Centers for Disease Control and Prevention Screening for *Candida auris* Colonization. https://www.cdc.gov/fungal/candida-auris/c-auris-screening.html.

[309] Aldejohann A.M., Wiese-Posselt M., Gastmeier P., Kurzai O. (2022). Expert recommendations for prevention and management of *Candida auris* transmission. Mycoses.

[310] Cadnum J.L., Shaikh A.A., Piedrahita C.T., Sankar T., Jencson A.L., Larkin E.L., Ghannoum M.A., Donskey C.J. (2017). Effectiveness of Disinfectants Against *Candida auris* and Other Candida Species. Infect. Control Hosp. Epidemiol..

[311] Ku T.S.N., Walraven C.J., Lee S.A. (2018). *Candida auris*: Disinfectants and Implications for Infection Control. Front. Microbiol..

[312] Rutala W.A., Kanamori H., Gergen M.F., Sickbert-Bennett E.E., Weber D.J. (2019). Susceptibility of *Candida auris* and *Candida albicans* to 21 germicides used in healthcare facilities. Infect. Control Hosp. Epidemiol..

[313] National Health and Medical Research Council (2019). Australian Guidelines for the Prevention and Control of Infection in Healthcare.

[314] Department of Health Estates and Facilities (2013). Health Building Note 00–09: Infection Control in the Built Environment.

[315] Tomblyn M., Chiller T., Einsele H., Gress R., Sepkowitz K., Storek J., Wingard J.R., Young J.A., Boeckh M.J., Center for International Blood and Marrow Research (2009). Guidelines for preventing infectious complications among hematopoietic cell transplantation recipients: A global perspective. Biol. Blood Marrow Transplant..

[316] Sehulster L.M., Chinn R.Y.W., Arduino M.J., Carpenter J., Donlan R., Ashford D., Besser R., Fields B., McNeil M.M., Whitney C. (2004). Guidelines for Environmental Infection Control in Health-Care Facilities. Recommendations from CDC and the Healthcare Infection Control Practices Advisory Committee (HICPAC).

[317] Raad I., Hanna H., Osting C., Hachem R., Umphrey J., Tarrand J., Kantarjian H., Bodey G.P. (2002). Masking of neutropenic patients on transport from hospital rooms is associated with a decrease in nosocomial aspergillosis during construction. Infect. Control Hosp. Epidemiol..

[318] Teh B.W., Yeoh D.K., Haeusler G.M., Yannakou C.K., Fleming S., Lindsay J., Slavin M.A., Australasian Antifungal Guidelines Steering Committee (2021). Consensus guidelines for antifungal prophylaxis in haematological malignancy and haemopoietic stem cell transplantation, 2021. Intern. Med. J..

[319] Centres for Disease Control and Prevention Targeted Environmental Investigation Checklist for Outbreaks of Invasive Infections Caused by Environmental Fungi (e.g., *Aspergillus*, *Mucormycetes*). https://www.cdc.gov/fungal/pdf/targeted-environmental-investigation-checklist-508.pdf.

[320] Infection Prevention and Control for *Candida auris*. https://www.cdc.gov/fungal/candida-auris/c-auris-infection-control.html#:~:text=Environmental%20sampling%20is%20generally%20not,auris.

[321] O’Loughlin M.C., Turner K.D., Turner K.M., Gupta V.K., Tuohy M.G., Ayyachamy M., Turner K.M., O’Donovan A. (2013). Quantitative Sampling Methods for the Analysis of Fungi: Air Sampling. Laboratory Protocols in Fungal Biology: Current Methods in Fungal Biology.

[322] Lacey J. (1991). Aggregation of spores and its effect on aerodynamic behaviour. Grana.

[323] Turgeman T., Shatil-Cohen A., Moshelion M., Teper-Bamnolker P., Skory C.D., Lichter A., Eshel D. (2016). The Role of Aquaporins in pH-Dependent Germination of *Rhizopus delemar* Spores. PLoS ONE.

[324] Kwon-Chung K.J., Sugui J.A. (2013). *Aspergillus* fumigatus—What makes the species a ubiquitous human fungal pathogen?. PLoS Pathog..

[325] Valsecchi I., Dupres V., Stephen-Victor E., Guijarro J.I., Gibbons J., Beau R., Bayry J., Coppee J.Y., Lafont F., Latge J.P. (2017). Role of Hydrophobins in *Aspergillus* fumigatus. J. Fungi.

[326] Napoli C., Marcotrigiano V., Montagna M.T. (2012). Air sampling procedures to evaluate microbial contamination: A comparison between active and passive methods in operating theatres. BMC Public Health.

[327] Łukaszuk C., Krajewska-Kułak E., Guzowski A., Kułak W., Kraszyńska B. (2017). Comparison of the Results of Studies of Air Pollution Fungi Using the SAS Super 100, MAS 100, and Air IDEAL. Int. J. Environ. Res. Public Health.

[328] Mould Testing Laboratory—NATA Accredited. https://www.sesa.com.au/mould-testing-laboratory-nata-accredited/.

[329] Anaissie E.J., Stratton S.L., Dignani M.C., Lee C.K., Summerbell R.C., Rex J.H., Monson T.P., Walsh T.J. (2003). Pathogenic molds (including *Aspergillus* species) in hospital water distribution systems: A 3-year prospective study and clinical implications for patients with hematologic malignancies. Blood.

[330] Hayette M.P., Christiaens G., Mutsers J., Barbier C., Huynen P., Melin P., de Mol P. (2010). Filamentous fungi recovered from the water distribution system of a Belgian university hospital. Med. Mycol..

[331] Lestrade P.P., Bentvelsen R.G., Schauwvlieghe A., Schalekamp S., van der Velden W., Kuiper E.J., van Paassen J., van der Hoven B., van der Lee H.A., Melchers W.J.G. (2019). Voriconazole Resistance and Mortality in Invasive Aspergillosis: A Multicenter Retrospective Cohort Study. Clin. Infect. Dis..

[332] Chang C.C., Hall V., Cooper C., Grigoriadis G., Beardsley J., Sorrell T.C., Heath C.H., the Australasian Antifungal Guidelines Steering Committee (2021). Consensus guidelines for the diagnosis and management of cryptococcosis and rare yeast infections in the haematology/oncology setting, 2021. Intern. Med. J..

[333] Douglas A.P., Smibert O.C., Bajel A., Halliday C.L., Lavee O., McMullan B., Yong M.K., van Hal S.J., Chen S.C., the Australasian Antifungal Guidelines Steering Committee (2021). Consensus guidelines for the diagnosis and management of invasive aspergillosis, 2021. Intern. Med. J..

[334] Hoenigl M., Salmanton-Garcia J., Walsh T.J., Nucci M., Neoh C.F., Jenks J.D., Lackner M., Sprute R., Al-Hatmi A.M.S., Bassetti M. (2021). Global guideline for the diagnosis and management of rare mould infections: An initiative of the European Confederation of Medical Mycology in cooperation with the International Society for Human and Animal Mycology and the American Society for Microbiology. Lancet Infect. Dis..

[335] Keighley C., Cooley L., Morris A.J., Ritchie D., Clark J.E., Boan P., Worth L.J., the Australasian Antifungal Guidelines Steering Committee (2021). Consensus guidelines for the diagnosis and management of invasive candidiasis in haematology, oncology and intensive care settings, 2021. Intern. Med. J..

[336] Aslam S., Rotstein C., on behalf of the AST Infectious Disease Community of Practice (2019). *Candida* infections in solid organ transplantation: Guidelines from the American Society of Transplantation Infectious Diseases Community of Practice. Clin. Transplant..

[337] Husain S., Sole A., Alexander B.D., Aslam S., Avery R., Benden C., Billaud E.M., Chambers D., Danziger-Isakov L., Fedson S. (2016). The 2015 International Society for Heart and Lung Transplantation Guidelines for the management of fungal infections in mechanical circulatory support and cardiothoracic organ transplant recipients: Executive summary. J. Heart Lung Transplant..

[338] Pappas P.G., Kauffman C.A., Andes D.R., Clancy C.J., Marr K.A., Ostrosky-Zeichner L., Reboli A.C., Schuster M.G., Vazquez J.A., Walsh T.J. (2016). Clinical Practice Guideline for the Management of Candidiasis: 2016 Update by the Infectious Diseases Society of America. Clin. Infect. Dis..

[339] Tissot F., Agrawal S., Pagano L., Petrikkos G., Groll A.H., Skiada A., Lass-Florl C., Calandra T., Viscoli C., Herbrecht R. (2017). ECIL-6 guidelines for the treatment of invasive candidiasis, aspergillosis and mucormycosis in leukemia and hematopoietic stem cell transplant patients. Haematologica.

[340] Ullmann A.J., Akova M., Herbrecht R., Viscoli C., Arendrup M.C., Arikan-Akdagli S., Bassetti M., Bille J., Calandra T., Castagnola E. (2012). ESCMID* guideline for the diagnosis and management of Candida diseases 2012: Adults with haematological malignancies and after haematopoietic stem cell transplantation (HCT). Clin. Microbiol. Infect..

[341] Cooley L., Dendle C., Wolf J., Teh B.W., Chen S.C., Boutlis C., Thursky K.A. (2014). Consensus guidelines for diagnosis, prophylaxis and management of *Pneumocystis jirovecii* pneumonia in patients with haematological and solid malignancies, 2014. Intern. Med. J..

[342] Helweg-Larsen J., Benfield T., Atzori C., Miller R.F. (2009). Clinical efficacy of first- and second-line treatments for HIV-associated *Pneumocystis jirovecii* pneumonia: A tri-centre cohort study. J. Antimicrob. Chemother..

